# One Health Evaluation: A Case Study at the University of Bologna

**DOI:** 10.3389/fpubh.2021.661490

**Published:** 2021-07-28

**Authors:** Maurizio Aragrande, Massimo Canali, Mariana Roccaro, Elisabetta Ferraro, Alessandra Bonoli, Federica Savini, Silvia Piva, Laura Gallina, Angelo Peli, Vittorio Sambri, Alessandra Scagliarini

**Affiliations:** ^1^Department of Agricultural and Food Sciences, University of Bologna, Bologna, Italy; ^2^Department of Veterinary Medical Sciences, University of Bologna, Bologna, Italy; ^3^Department of Civil, Chemical, Environmental and Materials Engineering, University of Bologna, Bologna, Italy; ^4^Department of Experimental, Diagnostic and Specialty Medicine, University of Bologna, Bologna, Italy

**Keywords:** one health, self-evaluation, theory of change, system thinking, interdisciplinarity, transdisciplinarity

## Abstract

The level of One Health (OH), or “One Health-ness,” of health interventions has been defined as the capacity to operate according to six dimensions concerning OH operations and OH infrastructures, respectively (thinking, planning, and working; and information sharing, reciprocal learning, and systemic organization). Although health initiatives and research increasingly claim their orientation toward OH, such a capacity is rarely assessed. The objective of this study is to evaluate the One Health-ness of the academic team of the University of Bologna (UNIBO Team) working in the “ELEPHANT” project (Empowering universities' Learning and rEsearch caPacities in the one Health Approach for the maNagement of animals at the wildlife, livestock and human interface in SouTh Africa). This project involves universities, six from South Africa and two from Europe, and aims at embedding OH in research and learning to enable the control of diseases at the human, animal, and environmental interface, and to emphasize the interests of local African communities with wildlife conservation. The methodology adopts the NEOH method, developed in 2018 by the EU-COST Action, “Network for the Evaluation of One Health.” The approach is based on questionnaires delivered to participants, which focus on the six OH dimensions, and then translate answers into quantitative metrics through the OH Index (OHI) and the OH Ratio (OHR). The following two evaluation levels are foreseen: the whole project and the single partner institutions. The evaluations are carried on in parallel, with preliminary, mid-term, and final assessments, to monitor the efficacy of the project actions. The preliminary evaluation of the UNIBO Team resulted in the OHI of 0.23 and the OHR of 1.69 which indicate a low degree of OH-ness and an imbalance between OH operation and OH infrastructure. The UNIBO case study will be the baseline for the evaluation of the other partner institutions involved in the ELEPHANT project. This type of evaluation can support the implementation of OH practices inside a project and underpin the strategies that allow to achieving more effective results. Any improvement in the OH-ness of each single academic team can be also considered as a result of the ELEPHANT project, thus showing its multiplier effect in the context.

## Introduction

The degree of One Health (OH-ness) is about the effectiveness of a project (of a team, an initiative) to materially operate according to six dimensions which identify to what extent it complies with One Health (OH) concept. These dimensions describe the adequacy of OH operations (i.e., thinking, planning, and working) and OH infrastructures (information sharing, reciprocal learning, and systemic organization). The OH concept requires system vision (1), interdisciplinary and transdisciplinary approaches (2, 3) to face and deal with the health challenges that arise from the interaction between humans, animals, and the environment. The approach of OH has been gaining increasing attention over the years (4, 5). It is argued that everyone would benefit from mainstreaming the OH approach as it seeks to maximize the benefit from interventions in the health sector as a whole (6). Indeed, this integrated approach has been promoted by international organizations to improve public health (7, 8). Implementing OH necessarily requires knowledge integration and working collaboration among scientists, as well as the engagement of multiple sectors (NGOs, local communities, and policymakers). However, some scholars warned that institutional constraints might preclude the practical implementation and evaluation of OH: academics and social actors often do not coordinate their actions, disagreeing on the causes, consequences, and problem-solving strategies of problems (9, 10). Moreover, little has been done to prove the efficacy of such opinions and criticisms, and the lack of standardized OH metrics has precluded the production of objective evidence on the potential benefits of this approach. In turn, this made policymakers and governments less prone and discouraged to put in practice the OH approach (4, 11). Many self-declared OH initiatives show little consistency and cohesion in planning and reporting their interdisciplinary efforts; few papers report the results of the collaborative processes, and the real OH outcomes appear difficult to characterize and identify/evaluate (11–14).

The Network for the Evaluation of One Health (NEOH; http://neoh.onehealthglobal.net/), a 2014–2018 European cooperation in science and technology (COST) Action, developed the evaluation framework to assess the level of OH-ness of a health initiative, based on the dimensions mentioned above that can affect the outcomes (15–20).

The Esarmus + Capacity Building project named ELEPHANT (Empowering universities' Learning and rEsearch caPacities in the one Health Approach for the maNagement of animals at the wildlife, livestock and human interface in SouTh Africa), kicked off in February 2020, aims at contributing to poverty relief through the enhancement of Southern African Higher Education Institutions (SA HEIs) by improving their innovation and research capacities. It adopts the OH approach implementing system thinking and interdisciplinarity and transdisciplinarity to develop more effective health management practices for humans, as well as wild and domestic animals. The project involves two European Universities (the Universities of Utrecht and Bologna) and six SA HEIs (the Universities of Pretoria, Limpopo, Fort Hare, Mpumalanga, Venda, and the South African Wildlife College), as well as five South African non-academic partners (AfriVet, the National Health Animal Forum, the National Institute of Communicable Diseases, the governmental Department of Science and Technology, and the Department of Agriculture, Forestry, and Fisheries) and one Dutch partner (Smart Parks). The project includes the evaluation of OH-ness, initially focusing on the individual partners and subsequently on the network as a whole. The ELEPHANT project applies the NEOH methodologies to self-assess its impacts through preliminary, mid-term, and final evaluations. The implementation of the ELEPHANT project is expected to change the way partners and stakeholders operate, by upgrading their approach to health problems using inter-/transdisciplinary and systemic practices, thus increasing their level of OH-ness. The OH-ness evaluation performed throughout the project will measure the evolution of the teams toward a greater degree of OH-ness, which may become a structural feature of the partner institutions. The partner team evaluation exactly aims at assessing whether and to what extent the participation of each partner team in the OH project entails an improvement of the degree of OH-ness of the partner institutions in activities that are independent of those developed in the ELEPHANT project.

The objective of this paper is to present the results of the preliminary OH-ness self-evaluation of the University of Bologna Research Team involved in the ELEPHANT project.

## Materials and Methods

As mentioned above, the OH-ness evaluation is based on a protocol developed by the NEOH through a wide consultation among the NEOH members and through a participative approach. The OH-ness evaluation method is a general evaluation framework that can be applied to any initiative related to the main sectors of OH (animal health, human health, and the environment), and whenever the complexity of a given problem is to be understood. In this occurrence, system thinking, interdisciplinarity and transdisciplinarity show their effectiveness in relation to traditional conceptual schemes. The evaluation is structured around the following four elements: (i) description of the activity and its context; (ii) description of the system and the theory of change (TOC); (iii) assessment of the OH-ness; and (iv) comparison of the degree of OH-ness and the outcomes produced.

The evaluation exercises developed so far focused on specific initiatives, such as research projects (21), individual human and animal health measures (22), and surveillance systems (23, 24). Moreover, these exercises were developed ex-post (even retrospectively in some cases) (15). The NEOH framework was adapted to the ELEPHANT project in the following two ways: (i) the evaluation process extends over three rounds of assessment (i.e., preliminary, mid-term, and final); and (ii) it is developed at both the project level and at each individual partner institution level. Partner team evaluations focus on the degree of OH-ness each institutional team involved in the ELEPHANT project achieved during the project development. In this case, the preliminary evaluation covers the activities developed by the partner teams before and independently from the ELEPHANT (i.e., before January 2020).

This approach required the NEOH protocol to be adapted to specific situations, in particular for identifying the teams and the activities undertaken and shared independently from the ELEPHANT project at any round of the evaluation. The OH-ness evaluation (at both the project and the partner team level) revolved around three elements. Element 1 and 2 are mainly based on narratives and visual representation tools. Element 3 is based on self-evaluation questionnaires delivered to the team members, allowing for quantitative or qualitative answers (the latter to be translated in quantitative metrics. The degree of OH-ness, calculated in Element 3, is then synthesized in two numerical indices, the OH-index (OHI) and the OH-ratio (OHR), which focus on the relevant dimensions of OH. The researchers of UNIBO met to prepare the submission of the ELEPHANT project in mid-2019 for the first time. Then, they started working as a team after the approval of the project, which kicked off in mid-February 2020. The UNIBO evaluation process was entirely shared with and participated by the team members *via* online meetings held from April to October 2020. According to the evaluation plan set by the ELEPHANT project, the evaluation of UNIBO is meant to open the way to evaluations at the level of partner institutions while fine-tuning the methodology in a participatory way in view of the evaluation of the whole project that follows a planned timeline (the second and third, i.e., final, evaluation rounds were planned, respectively at 20–24 and 34–36 months. The emergence of COVID-19 in the partner countries has led to delays in the evaluation timeline).

### Description of The UNIBO Team

Following the partner institution criteria, the description of the team and the activities of the members were included in Element 1. The team is composed of ten scholars and researchers working in different disciplinary domains (veterinary medicine, human medicine, agro-food economics, and engineering) belonging to four different departments of the University of Bologna: the Department of Veterinary Medical Sciences—DIMEVET (n = 5), the Department of Experimental, Diagnostic, and Specialty Medicine—DIMES (n = 2), the Department of Agricultural and Food Sciences—DISTAL (n = 2), and the Department of Civil, Chemical, Environmental, and Materials Engineering—DICAM (n = 1).

In order to identify and describe the team, each team member was asked to answer 10 preliminary questions about the team structure and activities. The latter were to be selected among the activities shared in practice with other team members and described by answering 12 specific questions. In particular, the team members were asked to give insights about the drivers, the objectives, the role in the context, the processes, and the expected results of the selected initiatives and their relevance to the OH.

### Description of The System and TOC

The description of the system and the TOC of the UNIBO team constitute Element 2. The initiatives are carried out in the context of the general environment in which the said initiatives developed by the team members operate and produce outputs, outcomes, and impacts. Following the paradigm of the system approach, the description of the context allows for the understanding of the system around the initiatives which includes the basic units (actors, institutions, and processes), their relationship and potential mutual influences (feedback, loops, synergies, and/or antagonisms), and results (output, expected and unexpected outcomes, and impacts). The description of the context is developed through a participatory, iterative, and interdisciplinary process. On the other side, the TOC outlines the way the activities started by the team members result in effects across a timeline by highlighting its underlying mechanisms (i.e., the cause-to-effect chains). The relevant processes are identified, including inputs (the resources used), outputs (the tangible products of the processes), as well as the way, in turn, the outputs produce outcomes (immaterial effects) and impacts (general effects within the limits of the system). Among the many activities implemented by the UNIBO team members, scientific research was selected as the main focus of the evaluation. In particular, despite low operational integration, team members converge on the awareness of problems and opportunities concerning the research on OH, which in turn inspires a shared TOC.

### Assessment of The OH-ness

The OH-ness assessment (Element 3) was performed *via* online meetings involving the whole team. The team was asked to answer specific questions based on the information retrieved from the preliminary steps of the process (team description, identification of the context, and the TOC). The team members answered the questions by sharing and discussing their viewpoints and perspectives, and a full consensus was obtained before giving the answers. For each answer, a score ranging between 0 and 1 was assigned. The features of Element 3 were then computed by using a Supplementary Microsoft Excel File, modeled on a template created by the NEOH. The resulting degree of OH-ness is divided into six main dimensions, each one assessing the various OH operations and infrastructures of the team. Following is a list of the dimensions, their meaning, and articulation.

#### Thinking

Thinking considers how the initiative conceives the system in which it operates and how far it ponders features that characterize complex adaptive systems. The OH Thinking is based on system thinking, which is defined as a framework of thought that is useful to holistically deal with complex things (25).

#### Planning

Planning consists of unfolding the OH Thinking into actions and operational features to obtain the expected goals of the OH initiative.

#### Working

One Health working focuses on the usual practices and work routines developed inside the team; these lead to the interdisciplinary and participatory engagement of the team within the initiative. In OH initiatives, data and information sharing often represent the staple that will contribute to a better comprehension of phenomena and a more inclusive and sustainable way of dealing with the challenge at hand.

#### Sharing

The sharing dimension deals with the protocols and facilities adopted by the team to ease information storage and access, methods, and results of the activity.

#### Learning

The learning dimension focuses on the level of learning that occurs within the team, the type of organization, protocols, or facilities that are put in place to serve this operation, and to what end learning is directed (modifying behaviors, changing team routines, or affirm values and paradigms). The dimension of “Learning” implies a change in cognition, potential, or actual behavior through better knowledge and understanding. It also includes exchanges at different levels (individual, team, and organization levels) between the team and the stakeholders (direct learning) and between the team and the external general environment.

#### Systemic Organization

Systemic organization is centered to assess the type of management and leadership of the team, for example, how competent and supportive leaders are in managing the team and pursuing OH objectives. Table 1 contains the details of the main attributes evaluated for each OH dimension.

**Table 1:** OH dimensions and their articulation.

	**OH dimension**	**Relevant attributes of the dimension**
OH operations	Thinking	1) System dimensions considered by the initiative and their balance, 2) correspondence between the dimensions of initiative and context, 3) system features, 4) TOC factors, 5) OH specific factors, and 6) social perspectives.
	Planning	1) Common aims, 2) stakeholder and actor engagement, 3) self-assessment and plan revisions, and 4) objectives.
	Working	1) Broadness of the initiative, 2) integration and collaboration, 3) transdisciplinary balance, 4) cultural and social balance, and 5) flexibility and adaptation.
OH infrastructures	Sharing	1) Sharing of general information and awareness, 2) data and information sharing, 3) methods and results sharing, 4) institutional memory and resilience.
	Learning	1) Individual learning, 2) team learning, 3) organizational learning, 4) direct learning, and 5) general environment learning.
	Systemic organization	1) Team structures, 2) management and leadership, 3) competence, and 4) innovation and OH orientation.

### Comparison of The Degree of OH-ness and Outcomes Produced

Once all the data and information about the six dimensions of OH are collected, the six dimensions are visualized in a spider diagram, in which each score (ranging from 0 to 1) constitutes a spoke. The diagram represents the operational aspects of OH (i.e., OH Thinking, OH Planning, and OH Working), and the complementary supporting infrastructures (i.e., Sharing, Learning, and Systemic Organization). Therefore, the surface of the diagram pictures the level of each OH dimension, reported by the OHI), which ranks from 0 to 1, (respectively the lowest and the highest degree of OH-ness), and the OHR, that assesses the balance between OH operations and OH infrastructure (see Supplementary Materials for details).

The premises of the partner team evaluation (i.e., the fact that most researchers formed a team for the purpose of the ELEPHANT project) represented a methodological issue in evaluating some of the OH dimensions because the team did not exist before the ELEPHANT project. In order to solve this problem (i.e., how to score the evaluation elements that assume the existence of a structured team), the team decided to assign a score to each subgroup within the team (i.e., corresponding to disciplines or departments) for the attribute or character to be evaluated, then calculate the average score of the subgroups within the team and to use this value as the score of the team. The integration of the team can be assumed by computing the integration index (I), which can be conceived as a measure of the structure of the team:

$$\begin{array}{l} I=\;\Sigma C/MaxC \end{array}$$

Where *C* is the number of relationships of each unit with the others, *MaxC* = ΣU* (Σ*U* – 1) is the maximum number of potential relationships within the team, *U* is the number of units (e.g., departments or other organizations) taking part in the team. The *I* value can span from 0 (no integration) to 1 (complete integration).

## Results

### The UNIBO Team and Its Context

The team was constituted in view of the ELEPHANT Project; therefore, before joining the Project, the team members did not operate as a group, but rather as individuals or subgroups. For this reason, the team members were divided into four subgroups according to their department affiliations, and each subgroup was coherent and relatively homogeneous within itself. Actually, all members shared OH awareness, but they did not share any other common process. Indeed, they mainly worked within their department, or with other researchers outside the reach and scope of the ELEPHANT Project. Before the launch of the ELEPHANT project, the researchers of the subgroups were involved in activities that included only few members of the wider team, either because they were not aware of the activities of each other in this field, or because they were simply involved in different activities. This is why the team showed a low integration degree (Integration Index = 0.167); indeed, the assessment of this parameter was carried out at the early stages of their cooperation. Regardless, the entire team shared awareness of the OH issues and the practice of OH-oriented methodological approaches, and valued its scientific and operational complementarity, as it is a multidisciplinary team. Having a common leadership seemed less important, even if it was perceived informally by the team members.

Each team member was involved in several different activities, but research alongside teaching was acknowledged as the one that brought together everyone within the team. Other activities, which might be carried out by individual research groups, were represented by dissemination projects and participatory activities, as well as other initiatives, such as social engagement and fundraising. Some members were also involved in the evaluation of health measures and other initiatives were put in place to obtain knowledge supporting evidence-based policies. Several members were involved in the knowledge and technology transfer processes and in the strategy design. The aforementioned activities are ongoing and expected to last for several years. The UNIBO team deeply recognized the need for a transdisciplinary and interdisciplinary approach and acknowledged the importance of an active participation of communities and stakeholders in OH-related activities to gain social acceptance. Therefore, team members and subgroups independently started to develop their research based on OH concepts, acknowledging that a holistic vision is crucial to approach and manage health problems. Their research ambitions and developments are aligned with the University of Bologna strategic plan 2019–2021 inspired by the 2030 United Nations Sustainable Development Goals (see the institutional site of the University of Bologna at https://site.unibo.it/almagoals/en).

The whole team was expected to obtain scientific evidence and systematically collected data as a basis for the formulation and writing of evidence-based policies with the goal of putting effective health strategies in place. The team was also involved in designing science-based methodologies toward disease preparedness. In particular, the team pursued the following: (i) the reduction of human and animal diseases through the development of innovative diagnostic and therapeutic tools, biohazard identification in food safety (e.g., antimicrobial resistance—AMR); (ii) the improvement of animal welfare by the identification of risk factors that can hamper both animal welfare (e.g., heat stress), animal health, and biosecurity; (iii) the reduction of environmental pollution and exploitation through research on waste management, the enhancement and protection of water resources, and the reuse of building materials to create zero-impact cities; and (iv) socio-economic welfare through the study of environmental and societal costs determined by zoonotic and non-zoonotic diseases, and specific OH problems (e.g., AMR) across sectors and societies (animal production and health sectors and the society) to strive for allocating resources in the most efficient way possible. Team members had already translated scientific knowledge into tangible results, but they recognized the need to embrace an interdisciplinary way of thinking and working to acquire a holistic perspective toward health challenges.

The team was involved in research processes to pursue the aforementioned aims. These processes may be summarized as follows:

- Study of disease dynamics and determinants (internal and external) at the human–animal environment interface
- Impact of diseases and disease management strategies at socio-economic and environmental levels.

These processes were partially integrated, as the study of disease dynamics is articulated in different disciplinary sub-processes that are considered as integrated. However, the study of disease impact at the socio-economic and environmental level was not integrated with the former at the time of the evaluation.

The team identified as process inputs all the disciplinary competencies (including interdisciplinary working methods), but also funding from different institutional bodies, infrastructures, and organizations of the University of Bologna (buildings, labs, IT equipment, communication, and institutional support). The projects of the team are mainly funded by regional, national, international/supranational organizations, and their dissemination initiatives and publications are mainly targeted to the international scientific community. Finally, considering the description of the team, the team members agreed on focusing the OH evaluation on the scientific research.

### The System and TOC

**Figure 1:**
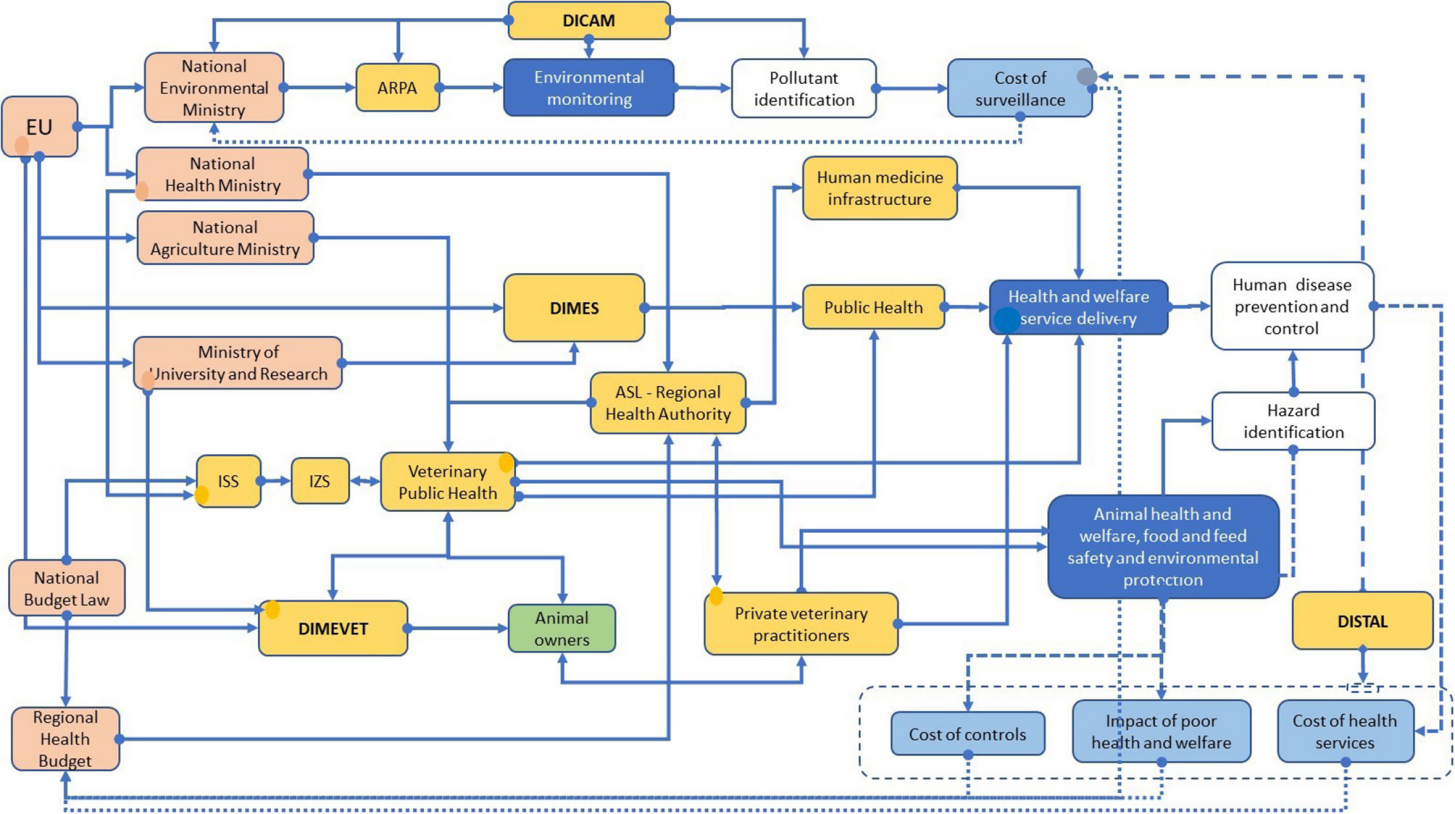
The system diagram of the UNIBO team.

In order to describe the system, team members started figuring their departments as elementary units in an empty space, describing their roles, activities, and outputs in relation to health problems. Then they internally investigated the relationships with other units, including their roles and activities. The reiteration of this approach allowed them to go larger and deeper in the description of the context. The result of this exercise is the diagram in Figure 1. The system diagram outlines the main decision-making organizations [EU, Italian Health Ministry, Italian Environment Ministry, Italian Agriculture Ministry, Istituto Superiore di Sanità (ISS), and Regional Health Authorities] and their functional relationships. These institutional bodies design the general health policy strategies and framework (rules and standards) at the EU and at the national level. National authorities transfer this information to regional authorities together with financial resources (as encoded in the national budget law). Based on their autonomy, the regional authorities organize the delivery of public health services. In the same way, environmental monitoring is carried out by ARPA, the regional authority for environmental protection. Competencies are shared between animal and human health scientists within the same regional organization. In particular, animal health researchers operate in close synergy with the national system of the Istituti Zooprofilattici Sperimentali (IZS) on the Italian territory and work under the mandate of the Ministry of Health. Hazard identification, as well as exposure assessment, characterize the surveillance activities performed by the IZSs and Aziende Sanitarie Locali *(*ASLs) (veterinary public health services) being aimed at designing the most effective sanitary measures to reduce the negative impacts on human and animal health.

Universities take part in the system through departments that cover specific research and teaching areas according to their competencies. Human and veterinary medicine departments, in particular, play specific roles in the health system as they are in charge of training private and public health professionals in human and veterinary medicine. Furthermore, their duty is to carry out research in different fields connected to health, provide scientific data, and develop innovative solutions to mitigate the impact of biological (pathogens) and nonbiological (pollutants) hazards on human, animal, and ecosystem health. Research activities in this field are financially supported by the EU Commission, the Ministry of University and Research, the Ministry of Agriculture, and the Ministry of Environment. Universities may be indirectly supported by the Ministry of Health when performing research with IZSs, ASL, ISS, and Istituti di Ricovero e Cura a Carattere Scientifico (IRCCS). University hospitals and Veterinary University Hospitals also play a key role in hazard surveillance by sharing data and competencies with local and national public health and veterinary public health services. Finally, the Ministry of Agriculture and the Ministry of Environment share competencies and strategies between them and the Ministry of Health. The ARPA works at the local/regional level in close cooperation with ASLs and refers to the Ministry of Environment; its duty is to identify and quantify potential environmental hazards that may directly or indirectly affect the health of humans, animals, and ecosystems. The role of the economic research in this context mainly consists in the evaluation of the cost of health policy measures (surveillance, health services, and distributional effects) which are meant to support the political decision; however, this type of information is poorly integrated into decision making and not systemically required (dashed lines in Figure 1 show this weak relationship). Finally, the team identified eight relevant system axes: geographical space, time, dimensions of life, knowledge creation, teaching, knowledge and technological transfer, economy, and social dimension (Supplementary Materials). These axes are the relevant dimensions which describe the functioning of the system.

**Figure 2:**
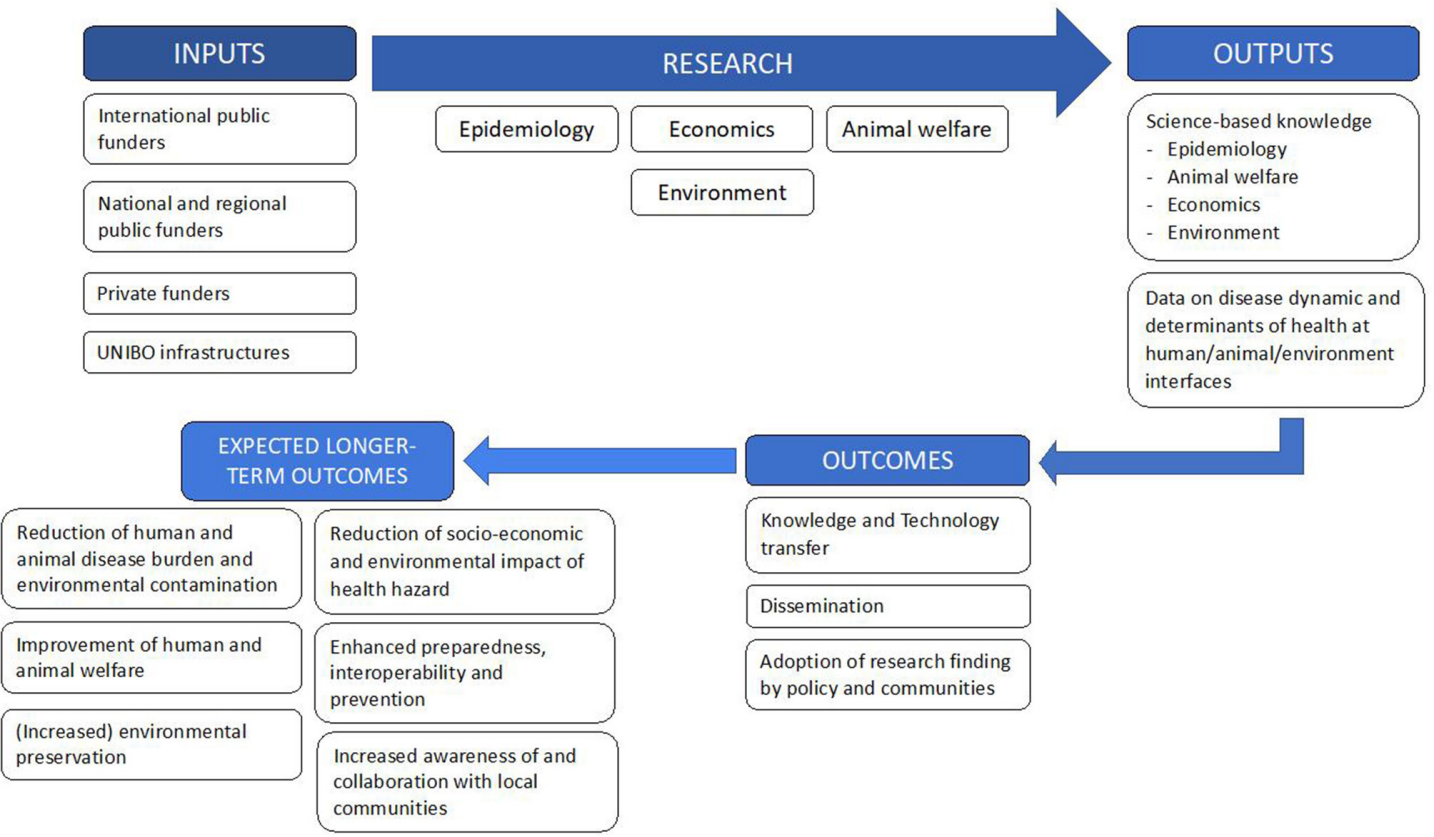
Theory of change (TOC) of the UNIBO team.

Concerning the TOC, the UNIBO team members converge on the awareness of the following problems and opportunities of the current research on OH: (i) the lack of knowledge about the complex relationships among usually separated sectors in the fields of health and their related sectors; (ii) the lack of operational methodology and practice to tackle wicked health-related problems; (iii) the possibility that, by filling those gaps, research may bring potential improvement of welfare across the society. Despite the low level of integration, all these elements provided an insightful input to team members at the moment of the evaluation. This shared awareness determines how researchers use resources and how they behave in order to get results and regulates their expectations as far as long-term impacts are concerned (Figure 2). As a consequence, a shared TOC can be drawn. Inputs are identified in the funds provided by regional, national, and international funders, both public and private. The infrastructures and organization of the UNIBO support the practical implementation of team activities or processes, that is, the research articulated in the following four main domains that closely reflect subgroup competencies: epidemiology, economics, animal welfare, and environment. Input and processes result in outputs or science-based knowledge in the mentioned fields and into data on disease dynamics and determinants of health at the human, animal, and environmental interface. Based on the acquired knowledge and data, the team expects to achieve outcomes identified by knowledge and technology transfer, dissemination among academic institutions and to society, and the embedding of research findings into policy and communities. Outcomes may be regarded as changes resulting from the initiative that can be considered as stepping stones to progress toward longer-term outcomes. Indeed, from these results, the longer-term goals of the team include the reduction of human and animal disease burden and environmental contamination, the improvement of human and animal welfare, increased environmental preservation, the reduction of the socio-economic and environmental impact of health hazards, enhanced preparedness, interoperability and prevention, and increased awareness of and collaboration with local communities.

As for system description, the description of the TOC will be used to evaluate the activities of the UNIBO.

System dimensions will be used in the assessment of the Thinking dimension of OH-ness to judge the ability of the team to apply system thinking in their activities and to comply with the underlying TOC in order to get the expected result (see Table 1).

### Evaluation of One Health-ness

**Figure 3:**
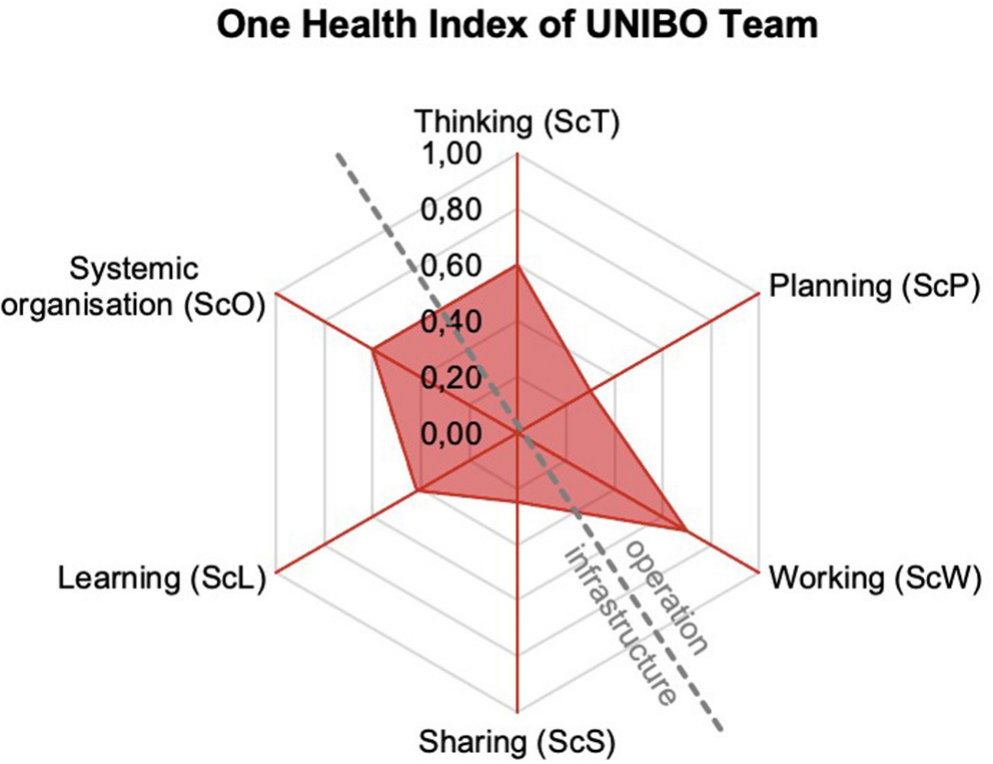
The NEOH spider diagram for the OH-ness evaluation of the UNIBO team. Spider diagram based on the overall scores (from 0 to 1, solid lines) of the six dimensions for the One Health Index (OHI) of the UNIBO Team (the red structure) from the One Health (OH) perspective. The dashed line represents the division of the diagram into operation and infrastructure for representing the One Health Ratio (OHR) of the UNIBO Team.

The overall OHI and OHR resulted as 0.23 and 1.69, respectively. The latter score is highlighted as a light imbalance between operations and supporting infrastructures. The OHI value of the UNIBO team reflects the surface of the red area in the spider diagram of Figure 3 and can be explained by the score obtained in each one of the six OH dimensions. Overall, the team has a low degree of OH-ness, which is especially influenced by the low scores in some dimensions and their articulation. The scores of the team in OH Thinking, OH Working, and Systemic Organization dimensions are relatively high (0.60, 0.70, and 0.60, respectively), but the ones related to OH Planning, Sharing, and Learning infrastructures scored low (0.30, 0.25, and 0.42, respectively). More insights stem from the articulation of the dimensions. Knowledge creation is considered by the UNIBO team as the most influential dimension of the system, as it is the main driver of the initiative itself, or even more as the springboard to obtaining specific means to intervene on health issues at different levels (human, animal, and environment). Besides, knowledge creation naturally ends in teaching, dissemination, and technological transfer, coherently with the outcomes of the TOC.

In general, the initiative established by the team can be considered as integrated, because it involves several levels of the system dimensions (e.g., life dimensions range from the microlevel of cells and micro-organisms to the macrolevel of the ecosystems and environment; the geographical space takes into account local, national, and international spaces; teaching includes all teaching cycles as well as life-long learning; economy includes the firm, farm, consumer behavior, markets, sectors, supply chains, society, and finance of the individuals). Regardless, these levels are approached independently by subgroups or individuals of the team as team members only partially act together. Therefore, even if the system is integrated, the team cannot be considered as equally integrated at this stage. This lack of coordination also reflects the embedding of activities in the TOC and their implementation. For instance, while the team does consider time dimension as a relevant aspect (as it influences disease effects and economics), timing and time delays are not considered in the TOC, as the team does not take actions to implement and speed up its research projects. Besides, research timing and time delays are often imposed by research funders, as team members have no control over them and are forced to respect them.

OH Planning scores 0.30. Despite the convergence toward common OH aims among subgroups, at the time of the evaluation, they developed their activity almost independently from each other without a common decision on their purposes, except for the team members affiliated to DIMEVET and DIMES, who partially design common goals. Indeed, the general environment and facilities of the main institution (the University of Bologna) promoted the convergence of individual groups toward a common aim (thanks to the information sharing, institutional routines), without the team deciding on that in advance or planning for it.

Moreover, the identification of actors and stakeholders as well as their engagement occur *ex facto*. This may happen, for instance, because of their financial support, and not according to a planned activity in the team or in the subgroups. Most research activities are funded by public or private funders and the budget is allocated for specific OH aims, but currently, there are no structured reiterative processes in place to critically revise the activities. Despite this fact, planning regularly happens through routine practices leading to result assessment and activity readdressing.

The overall score of OH Working dimension is 0.70. The initiative is wide both in its scope and in its disciplinary background, as the fields involved are several and diverse (veterinary and human medicine, epidemiology, animal production, environmental science, and economics). However, at this stage, disciplines are rarely integrated and embedded during the execution of research projects, although team members acknowledge the importance of an interdisciplinary approach. Furthermore, a transdisciplinary approach is sometimes applied by individual subgroups with no systematic application.

The overall score for Sharing infrastructures is 0.25. Before the ELEPHANT project, the team was not used to share protocols, routines, and resources of any kind; data and information were usually spread among team members using IT devices, such as chats and clouds. No specific funds are allocated to share facilities within the UNIBO team. The team benefits from the following data quality assurance: the mechanisms and procedures available at the University of Bologna are mainly focused on safeguarding data access, information, and results in case of system changes, such as institutional clouds, publication repository, and dedicated department to support IT production and consultancy.

The overall score for Learning infrastructures is 0.42. The team mostly embraces individual and team learning. Indeed, the team states that its direct environment (i.e., the information exchange between the team and the public and private stakeholders) is relevant to support both adaptive learning (i.e., the information applied to improve procedure) and generative learning (i.e., the information that can modify values and norms of the individuals). By the same token, the general environment (the culture, the economy, and the environment surrounding the team) is considered important by the team as it promotes adaptive and generative learning. Therefore, the team often learns how to improve and correct existing procedures, processes, and competencies, and its setting encourages it to look beyond the current situation.

The overall score for the Systemic Organization is 0.60. Teamwork is applied on a regular basis in each subgroup and among few of them. Due to the team structure, the teamwork does not represent a general feature of the team as a whole. This reflects the fact that, within the team, team management and team leadership are rarely officially identified, but they are effective in supporting the goals of the initiative. The leadership in the team complies with tasks, relationships, and changes. The team recognizes both central leadership, which inspires and promotes several activities, and decentralized ones at the level of subgroups. Thanks to this structure, there are no prevailing hierarchies in the initiative. All team members show open-mindedness and conceive themselves as able to bear and manage tensions. Competencies in various fields are allocated adequately toward the aspects of OH to gain new and relevant scientific knowledge.

## Discussion

The NEOH guidelines represent an original evaluation framework for the assessment of OH operations within an initiative and, so far, those guidelines have been used to assess the degree of OH-ness of specific health projects. Some of these projects are built up according to an OH perspective (17), some others are built up with holistic awareness of the system, even before the OH gained such recognition (26).

The aim of the OH-ness evaluation in the ELEPHANT project is different from the previous exercises and is 2-fold. The ELEPHANT project declares a strong OH orientation (as shown by the systemic and interdisciplinary conceptualization of health problems and by the transdisciplinary design of the project). In addition to the EU project evaluation standard, the project partners decided to develop the evaluation of the OH-ness across the whole duration of the project dividing it into three rounds. The project-level evaluation closely reflects the NEOH method; however, it is different from the evaluation exercises developed so far as it provides the opportunity to assess how the degree of OH-ness will evolve during the project implementation and to what extent this will be reflected in the behavior of the partner institutions in other activities outside the ELEPHANT project. On the other side, the aim of the team-level evaluation is to portray the evolution of each team toward the OH perspective throughout the years and to describe how the team will implement the application of such philosophy and way of operating. The underlying assumption was that the OH initiative like the ELEPHANT project, regardless of its aims and goals, could build upon and widen the actual way researchers think and act within the OH domains. Indeed, OH is not just a thematic research area, but rather a method of thinking and acting in order to deal with complex health issues. For this reason, it is not enough to study the OH topic, but it is rather a matter of how a problem is faced and studied, and the methodology applied. This is what OH means. Indeed, one of the key aspects of OH is collaboration, which should be enhanced among disciplines, academia, and societal and political parties.

The present study applied for the first time the NEOH evaluation framework to a group of researchers who joined the team to take part in the OH project and did not have any previously established collaborative relationships. The aim of the evaluation was to assess the degree of OH-ness of the UNIBO team before the ELEPHANT project was started, in order to highlight the weaknesses and strengths of the team in relation to the implementation of OH. From this evaluation, the team has the chance to put into practice corrective actions to implement OH collaborative working and operations. The evaluation also allows to portray a dynamic picture of the team: the evaluation will be repeated throughout the years of the ELEPHANT project and at its end in order to assess its expected outcomes (improving OH-ness) as well as unexpected ones in each partner institution, including the University of Bologna. Therefore, the results presented here should be considered as a starting point, from which to implement the OH operations.

As mentioned above, the UNIBO team was initially unintegrated or partially integrated. Indeed, researchers belong to different departments (DIMES, DIMEVET, DISTAL, and DICAM) and different scientific areas (veterinary medicine, medicine, engineering, biotechnology, and agro-food economics). DIMES and DIMEVET had already developed relationships throughout the years, either occasionally or on a systematic basis, for collaborations in research projects, or for other academic activities. The other researchers, belonging to DISTAL and DICAM, worked individually, or with other researchers than those involved in the ELEPHANT project. All the early subgroups of the team implemented research in the same institution (the University of Bologna) and dealt with OH for a relevant part of their research activity. This meant that they shared a similar vision about the methodological innovation brought in by OH (interdisciplinarity and transdisciplinarity and holistic or system approach, implementing them to some extent). However, they did not share common research activities before their involvement in the ELEPHANT project, nor they worked or planned to work together. This led to some trouble, while answering the questions and assigning the evaluation scores, because there were varying degrees of integration and collaboration among the team members. This query was resolved by pondering the answers given by subgroups and by calculating the (weighted) average score of the groups within the team and then using this value as the score of the team. However, the identification of the system and the TOC reflected the activity of all members, to the extent that all UNIBO researchers have common aims in relation to OH, even if they pursue their goals independently from each other and in different scientific domains.

The evaluation revealed an overall OHI of 0.23 (where the value 1.00 indicates the highest level of OH-ness) and the OHR of 1.69, showing a slight imbalance between OH operations and supporting infrastructures. The team scored relatively high in OH Thinking, OH Working, and Systemic Organization dimensions (0.60, 0.70, and 0.60, respectively), but scored low in OH Planning, Sharing, and Learning infrastructures (0.30, 0.25, and 0.42, respectively). The team demonstrates a general positive orientation toward the conceptual approach to OH (particularly system thinking, interdisciplinarity, dealing with specific OH problems) and seems currently lacking in OH infrastructures (sharing of information and procedures, mutual learning, and shared practices).

Overall, this evaluation pictures a team that is conscious and aware of all the opportunities brought in by the OH approach but needs to fully translate into real actions that it is able to conceive only partially. To put it simply, in order to solve complex problems, any kind of boundaries should be overcome. In order to take the advantage of the common OH consciousness, the team should identify and develop shared activities. Additionally, the team could put in place learning infrastructures that would allow evolving from an unintegrated situation to a collaborative-integrated OH team.

With this purpose, the team could establish a common organization, sharing facilities, protocols, and procedures. To achieve such a goal, the team should have a better-defined structure, and this could be obtained by refining role designation and responsibilities, ensuring that the competencies of team members are assigned to the goal of the initiatives and task development. The team needs to implement the engagement and the collaborative relationships with stakeholders, to go beyond the funding-subordination while concretely and actively involving the latter. It should make the effort to take a step further to develop a learning process that could allow the team not only to evolve but also to put into practice the principles of collaboration and transdisciplinarity. With a dynamic learning process that entrenches the attempt to manage the culture of that organization, the team should be able to discard old routines to make way for new ones. As a learning organization seeks new perspectives by taking risks, it creates a competitive advantage for the organization itself. Ultimately, experiments should be carried out responsibly and with calculated risk. Despite unavoidable failures or mistakes, the team should show acceptance in order to learn from such mistakes and retain the positive inquisitiveness of those experiments (27).

Finally, the team needs to start acting and evolving as a group during the course of the project. Indeed, the team started to be engaged in other projects and to put in place the multiple competencies to gain and acquire new knowledge. The results obtained in the OHI and OHR of this evaluation are not a static report, but rather a dynamic and evolving score and will be compared and further discussed in future subsequent evaluations.

## Limitations

The evaluation exercise of the UNIBO team allowed for testing the effectiveness of the NEOH approach. The first criticism might regard the objectivity of the method. For instance, system dimensions are identified by team members based on their perception, not on an objective list of dimensions. This hinders the possibility to compare the degree of OH-ness across cases, though still allowing for comparisons of OH-ness improvement across time concerning a team or a project. By the same token, it should be borne in mind that as a self-evaluation process, the UNIBO exercise may bear a subjectivity bias, resulting in potential over/under-scoring.

Secondly, the current version of the method does not assign specific weight to the dimensions, nor it accounts for individual divergencies, which remain implicit in the average values used to calculate the OH-ness indices. Accurate information on these aspects may lead to more effective diagnostics of the strengths and weaknesses of the team or project to support effective management. These are key points to design and implement OH-ness improvement strategies.

Finally, the ambition to formulate a quantitative index for OH-ness may lead to disregard the narrative of qualitative variables behind the quantification. Indeed, the way a narrative can be translated into a quantitative scoring deserves more consideration in view of improving the effectiveness of the evaluation method.

Some of the limitations outlined here were addressed by the UNIBO team. To avoid subjectivity biases, the team was engaged in several revisions of the scoring to improve their evaluation accuracy. This process was based on reiterative discussions and revised narratives which provided a background to the scoring process. Alongside OH-ness evaluation, the UNIBO team started a bibliometric evaluation of the degree of interdisciplinarity, transdisciplinarity, and collaborative habits of team members that will be the subject of the forthcoming publication. This will contribute to providing a more objective basis to transform the qualitative assessment of those aspects into a quantitative assessment based on scientific results.

Further limitations emerged while implementing the evaluation process that the team members perceived as complex and time-consuming. Indeed, they were asked to conceptualize their current research practice in ways that were new to them. Despite this, however, the exercise was considered very effective as it prompted a deep reflection on OH. The results of such reflection are summed up by the OHI and OHR and should be considered as a benchmark for further team development that will be measured through the next evaluation rounds planned along with the project development. Hopefully, further practice with the evaluation among the UNIBO team will lead them to suggest a plethora of solutions to improve the evaluation method itself, especially in terms of the above-mentioned limitation.

## Conclusion

The OH approach is increasingly gaining recognition in academia, research, and among governmental institutions, triggering the development of health policies. However, an ongoing debate about this concept still exists among scientists and scholars that may jeopardize the attempt to implement such a framework.

Indeed, OH is more than a declared concept: it is a way of acting toward more collaborative initiatives among human health, animal health, and environmental health specialists. Nevertheless, efforts to demonstrate the effectiveness of OH initiatives rarely occur or succeed in the scope. Thus, obtaining such information is vital for the implementation and the development of OH initiatives that could bring positive effects on the human/animal/ecosystem interface.

This study applied an innovative approach for the evaluation, under the OH lens, of research processes and working habits of the team of researchers from the University of Bologna. This approach (as an implementation of the NEOH method) resulted in the preliminary estimation of the OH-ness degree of the UNIBO Team participating in the ELEPHANT project. In turn, this created a benchmark for comparison with the next evaluation rounds that are envisaged by the ELEPHANT project.

The evaluated activities of the Unibo Team are independent of those that will be subsequently developed in the ELEPHANT project; for this reason, the future evaluation will provide a dynamic portrait of the expected outcomes within the ELEPHANT project in its partner institutions. For each team, this will be an opportunity for self-reflection and improvement toward the extensive implementation of OH as a concept and a philosophy. To date, such an evaluation has never been performed in academic institutions, but only in OH projects or in initiatives within OH-related reach and scopes.

## Data Availability Statement

The raw data supporting the conclusions of this article will be made available by the authors, without undue reservation.

## Ethics Statement

Ethical review and approval was not required for the study on human participants in accordance with the local legislation and institutional requirements. Written informed consent for participation was not required for this study in accordance with the national legislation and the institutional requirements.

## Author Contributions

MA, MC, and AS participated into the conceptualization of the paper and developed the conclusions. MA and EF drafted the manuscript. All authors participated into the discussion, contributed to reviewing, and approved the submitted version.

## Conflict of Interest

The authors declare that the research was conducted in the absence of any commercial or financial relationships that could be construed as a potential conflict of interest. The handling editor declared a past co-authorship with MA and MC.

## Publisher's Note

All claims expressed in this article are solely those of the authors and do not necessarily represent those of their affiliated organizations, or those of the publisher, the editors and the reviewers. Any product that may be evaluated in this article, or claim that may be made by its manufacturer, is not guaranteed or endorsed by the publisher.
