# Supplementary material for: One Health Evaluation: A Case Study at the University of Bologna
Source: Front Public Health. 2021 Jul 28;9:661490. doi: 10.3389/fpubh.2021.661490 (PMC8355810; doi:10.3389/fpubh.2021.661490)

Erasmus+

ELEPHANT Project

Questionnaire for the Evaluation of the One Health of Partner Institutions: UNIBO Team

(D R A F T)

August 2020

University of Bologna

ALMA MATER STUDIORUM
UNIVERSITÀ DI BOLOGNA

Developed by the UNIBO Team:
Aragrande M, Bonoli A, Canali M, Ferraro E, Gallina L, Peli A, Piva S, Roccaro M, Sambri V, Scagliarini A,

Structure of the document

Section 1: Description of the UNIBO Team

Section 2: Team initiative

Section 3: Description of the context

Section 4: Theory of change

Section 5: Evaluation of OH-ness

- 5.1) Thinking
- 5.2) Planning
- 5.3) Working
- 5.4) Sharing
- 5.5) Learning
- 5.6) Systemic organization
- 5.7) OH evaluation

List of tables

1.1 - Team description
2.1 - Description of the initiative
5.1 – Reference framework for graduation
5.2 – System dimensions
5.3 – Importance given to system dimension in the initiative
5.4 – Scales of the dimensions
5.5- Attributes of Thinking
5.6 – Scoring questions: Thinking
5.7 – Attributes of Planning
5.8 – Scoring questions: Planning
5.9 – Attributes of Working
5.10 - Scoring questions: Working
5.11 – Attributes of Scoring
5.12 – Scoring questions: Sharing
5.13 – Learning levels and types
5.14 – Attributes of Learning
5.15 - Scoring questions: Learning
5.16 - Attributes of Systemic organization
5.17 – Scoring questions: Systemic organization
5.18 – OH indexes

List of figures

3.1 – System description
4.1 - Theory of change
5.1 – Structure of the team

Questionnaire

This document implements the NEOH approach for the evaluation of One Health. It follows the guidelines contained in the *Conceptual framework for the evaluation of OH-ness* (see file).

Section 1: Description of the UNIBO Team

The UNIBO Team participating in ELEPHANT includes:

1. Alessandra Scagliarini, DVM PhD, DIMES
2. Vittorio Sambri, MD PhD, DIMES
3. Angelo Peli, DVM PhD, DIMEVET
4. Silvia Piva, DVM PhD, DIMEVET
5. Mariana Roccaro, DVM PhD, DIMEVET
6. Laura Gallina. Animal Biotech. PhD, DIMEVET
7. Elisabetta Ferraro, VET Student, DIMEVET
8. Maurizio Aragrande, Agro-food economist PhD, DISTAL
9. Massimo Canali, Agro-food economist PhD, DISTAL
10. Alessandra Bonoli, Engineer PhD (è corretto?), DICAM

Table 1.1 - Team description

1.	Nr of members taking part of the team	10	
2.	Nr of disciplines represented in the team	5	
	a. Count the nr of members per discipline	**Discipline**	**Nr of members**
		Veterinary Medicine	5
		Economics	2
		Biotechnology	1
		Medicine	1
		Engineering	1
	b. Count the nr of members per institutions/departments	**Institution/Dept**	**Nr of members**
		DIMEVET	5
		DIMES	2
		DISTAL	2
		DICAM	1
3.	Geographical representation of the team members?	**Geographical level**	**Nr of members**
		Local/regional	10
		National	
		International/European	
4.	Nr of stakeholders (1) represented in the team		
5.	What and how many different social instances (2) are represented in the team?	**Kind of institution (3)**	**Nr of members**
	➢ Social instance A	University	10
	b. Social instance B		N/A
	c. Social instance C		N/A
	d. …		
6.	What is/are the common motivation(s) of the team? *Assign a score to each aspect according to its importance in a scale from 0 (not relevant/not applicable) to 5 (very important)*	**Motivation**	**Score**
		The vision of a problem	5
		Scientific complementarity/synergy	4
		Operational complementarity/synergy	3
		Spatial proximity	0
		Organizational proximity	0

		Recognizing a common leadership	1
		The way of working	0
		Other (describe)	
7.	What activities are shared in practice?<br>*Assign a score to each aspect according to its importance in a scale from 0 (not relevant/not applicable) to 5 (very important)*	**Activity**	**Score**
		Research	5
		Teaching	4
		Dissemination	3
		Technology transfer	2
		Projects participation	3
		Management	1
		Social engagement	3
		Evaluation health measures	2
		Support decision making	2
		Strategy design	2
		Fund raising	3
		Other (describe)	
8.	How many members share the above-mentioned activities?<br>*E.g. 3 members share 2 activities; 4 members share 1 activity; etc.*	**Nr of activities**	**Nr members**
		1	9
		2	8
		3	4
		4	4
		5	3
9.	How long the team members are sharing the same activity?<br>*Score according to the weighted average time between team members and period of sharing the same initiative (e.g. 2 members shared the same initiative for 2 years and 1 member joined the initiative the second year = (2x2+1x1)/2 = 2.5 years). Time can be measured in years or year fractions*	**Nr of members**	**Time sharing**
		5	5 years
		4	5 years
		4	1,5 years
		2	6 years
10.	What is the time perspective of the team members in the activities? (4)<br>*Duration can be expressed exactly (years, months) or approximately (few months, 1 or few year, several years). This may reflect exact knowledge in the case of a project or just a feeling or wish. Please mark with a symbol or a sign the second case*	**Activity**	**Duration**
		Research	Several years
		Education	Several years
		Dissemination	Several years

Notes to Table 1

(1) Non-academic institutions, social bodies, NGO, professional associations, syndicates, government bodies, etc

(2) Cultural-ethical, religious, governmental, gender, health, political, animal, production sector

(3) Public, private, NGO, no profit, network, international organization (e.g. WHO, OIE, FAO, EU or similar)

(4) Duration is relevant as we aim to evaluate the OH-ness of the team in parallel with ELEPHANT duration (3 years). Changes in team participants may occur during this period, as well as the re-focusing of aims or way of working. This is not a problem for the evaluation as long as we want actually evaluate such dynamics occurring in the team as a collateral effect of ELEPHANT.

Section 2: Team initiative

Consider the answer to questions 7-10 of Table 1. Select a few activities (1 or 2) among those mentioned in the table. Criteria for the section of the activities should reflect the relevance for the OH, the highest possible level and time of engagement of the members as a team, and also the likely duration of the activity over the years to come (see note 4 to Table 1): i.e. the activity that involves the greatest number of participants for the longest time, or involves a relevant number of team members for the longest time. Ideally, it would be better to choose 1 activity. Otherwise each activity should be evaluated individually and this will multiply the need of time and resources for the evaluation. This said, focus the selected initiative(s). The questions in Table 2.1 provide some guidelines for the description and a list of issues which are relevant to understand what the initiative is. The expected result should be a narrative, possibly structured according to relevant issues such:

- Drivers

- Objectives
- Role in the context
- Processes
- Expected result
- Relevance for the OH

The evaluator can adapt the questions to the specific situation and the context (some aspects of the description might not be relevant or not be applicable to the specific initiative; others not included in the table might reveal fundamental). Keep in mind that a careful description of the initiative will provide useful elements, for the description of the context, the theory of change and to answer some evaluation questions concerning OH elements.

Overall description of UNIBO team approach to research answers to Table 2.1 narrative form:

Table 2.1 – Description of the initiative

**1) What is the selected initiative?**<br>*Name and describe the initiative in few lines to outline the main features and characteristics*
The selected activity is RESEARCH. Team members develop research in different domains which could hardly be defined by one thematic descriptor. Four main research fields can be identified which show relatively homogeneous features and/or aims (see point 4 below) among team members:<br>(a) Epidemiology: the research focuses on distal and proximal environmental risk factors of transmissible diseases, on farm biosecurity, setting up of disease control tools and anti-microbial resistance (AMR) in both domestic animals and wildlife populations, zoonotic diseases.<br>(b) Animal welfare: ruminant welfare assessment of farm and during transport, heat stress in dairy and beef cattle, welfare of the animals used for scientific purposes.<br>(c) Environment: development of a sustainable society, through the enhancement of natural resources, the reuse of building materials and waste management in the context of circular economy.<br>(d) Economics: evaluation of private and public costs and benefits emerging at the interface between animal, human, and environmental health. This adds economic information to decision making.<br>In general, research developed by team members is in line with the 2030 UN SDG goals, adopted by the University of Bologna as the main reference framework for its activities.

**2) Why does the initiative exist?**<br>*What are the reasons that determined the initiative? What does justify the existence of the initiative*
Health problems are complex and need a holistic vision that places specific research aspects in their context to effectively work in a One Health perspective. Social acceptance is greatly needed to the effectiveness of health measures, therefore an active participation of communities and stakeholders through a transdisciplinary approach is crucial. UNIBO team recognizes such needs and the lack of interdisciplinary and transdisciplinary connections in the production of science and knowledge. For these reasons the team members started to develop their research based on OH concepts.

**3) What are the aims of the selected initiative?**<br>*What does the initiative materially do or make? How does the initiative contribute to a wider problem or a general context? In particular, what problem does the initiative want to solve in that context?*
UNIBO team develops research in the above-mentioned domains with specific aims:<br>(a) Epidemiology: this research plays a role in expanding knowledge on AMR in animal populations, prevention and control of zoonotic diseases caused by viral, bacterial and parasitic agents, and developing innovative diagnostic and therapeutic tools. Research activities are also focused on biohazards affecting

food safety. Scientific results are translated into technologies and disseminated to the scientific community and society as a whole (social and public engagement).

(b) Animal welfare: research initiatives are focused on the assessment of the risk factors that can influence animal welfare and on farm biosecurity and welfare assessment in cattle and small ruminant farms; it is aimed at acquiring new knowledge and facilitating the development of evidence-based legislation that will also respond to the changing societal expectations. Research on heat stress is aimed at identifying the environmental and management risk factors, its impact on animal health and welfare and the possible mitigation strategies in order to cope with the climate change challenges, which affects ecosystems, biodiversity, water availability and food security.

The animal welfare group is involved in local, regional and national boards for the ethical and scientific evaluation of research projects in order to guarantee the quality and integrity of research; it also carries out research aimed at the study of low-neurological-development animal models and the development of alternative methods to animal experimentation, for a more ethically acceptable research.

(c) Environment: environmental research focuses on waste prevention and management, enhancement and protection of natural resources such as water and aquifer, and reuse of building and innovative materials, in order to design zero-impact buildings. It aims to develop the creation of sustainable cities and contribute to produce technologies for water supply, processing of wastewater and recycle of waste in developing countries. In addition, it contributes to draw up evidence-based policies.

(d) Economics: economic research focuses on the environmental and societal costs determined by zoonotic and non-zoonotic animal diseases, and specific OH problems (such as anti-microbial resistance) across sectors and society (animal production sector, health sector, the society). This contributes to rational, evidence-based choices in the implementation of health policies, which may save scarce resources. Economic research often integrates epidemiology and social sciences in an interdisciplinary approach.

We aim to get scientific evidence and systematically collected data as a basis for the formulation and writing of evidence-based policies to put in place effective health strategies. The team already translates science into tangible results for the benefit of the society; but we need to improve the integration of different scientific fields in order to acquire a holistic view of health problems.

We pursue the reduction of human and animal diseases and environmental pollution, as well as the improvement of human and animal welfare, keeping in mind that diseases are not only the indicator of human and animal welfare, but also of socio-economic welfare. We aim to work in wider context, considering that every particular situation can be different and that can have its own problems and own particularities, but we aim to produce scientific data that can be used transversally and in different circumstances.

4) How the initiative is meant to solve the targeted problem?
What are the specific actions that lead to the expected result? What processes are activated? How are those processes combined in view of the aim?

In order to pursue our goals, we activate research processes in different fields, such as epidemiology (e.g. microbiology, virology and implementation of innovative diagnostic tools), animal welfare, environment and economy (e.g. impact assessment of health measures). Two main research processes can be summarised as follows:

a. Study of disease dynamics and determinants (internal and external) at the human/animal/environment interface
b. Impact of diseases and disease management strategies at socio-economic and environmental levels

Research outputs from the above-mentioned processes are disseminated to the international academic community in order to increase the critical mass of knowledge. Research outputs are meant: - to provide the basis for further scientific development; - to provide scientific evidence to sustain material actions for

disease control; - by this way, to increase (human and animal) population welfare and environment preservation.<br><br>Processes are partially integrated: process (a) is articulated in different disciplinary sub-processes that can be considered as integrated; process (b) is not currently integrated with process (a).

**5) What is the timeline of the initiative?**<br>*Identify a starting and ending point, if applicable (e.g. a research project); or is it an iterative process, conditioned by result (e.g. a surveillance programme or a medical programme)? Is it continuous (e.g. a structured teaching programme)?*
Research activities are continuous. Most of the activities and processes started years ago and they are foreseen to last years in the future.

**6) What geographical level does the initiative involve or target?**
The geographical dimension of the research activities developed by team members covers several spatial levels, ranging from local to national and international. This also includes funding (coming from regional, national and international/supranational organizations), dissemination and publication (mainly international).

**7) What are the other relevant dimensions involved in the initiative?**<br>*Social sector, economy, life dimensions, organization, knowledge creation, teaching or training, dissemination*
Our research concerns life dimensions (such as microbiology, animal and human populations, the environment), knowledge creation, economy, dissemination, teaching, training, social dimension (through stakeholders' involvement).

**8) In which sectors of the OH does the initiative operate?**<br>*Human health, Animal health, Plant health, Environment*
The initiative operates at human/animal/environmental levels.

**9) At what level does the initiative operates?**
The initiative is aimed at creating specific knowledge to solve current health-related problems, identifying patterns and models (including impact models), designing scientific approach and methodology, including preparedness.

**10) How does the initiative influence OH sectors and/or their relationships?**
Research activities developed by the team provide useful results to improve specific aspects of OH. In some cases, results stem from integrated approaches and can/will provide highlights on the relationships among human, animal and environmental health (i.e. ROADMAP on AMR, …..). Anyway, this involves an individual domain or processes within the team but not the team as a whole or its relevant parts, as evidenced by the individual participation to national and international research projects.

**11) What kind of resources are in place to make the initiative work?**<br>*Competences, material and immaterial infrastructure, funding, specific equipment, …*

Process inputs are disciplinary competences (including interdisciplinary working methods), funding from different institutional sources (local, national, international), UNIBO organization and infrastructure (building, labs, IT equipment and communication, institutional support).

**12) How does the team develop the selected initiative(s)?**<br>*What is the degree of integration of the team in pursuing activity's aims? Is the initiative conceived according to a common plan? Are resources/processes coordinated?*
The research team, before its involvement in ELEPHANT, could be considered as structured in 4 small groups. These groups do share OH concept (as described above) but worked almost independently and separately. This is reflected by the two main processes identified under point 4. Actually, each group or process is relatively coherent and homogeneous in itself, but the groups did not share any common process.<br>*It should be underlined that the groups forming the team are not completely integrated in one team. If we consider the Departments, the groups belong to 4 departments (DICAM, DIMES, DIMEVET, DISTAL). Two out of these 4 departments (namely DIMES and DIMEVET) have regular relationships and contacts and can be considered integrated to some extent in the activity, the other 2 do not have relationship before ELEPHANT (see below Section 5).*

Section 3: Description of the context

The system diagram outlines the main decision-making organizations (EU, Italian Health Ministry, Italian Environment Ministry, Italian Agriculture Ministry, *Istituto Superiore di Sanità* -ISS-, Regional Health Authorities) and their relationships. These institutional bodies decide the general health policy strategy and framework (rules, standards) and the environmental strategies at the EU and National level. National authorities transfer this information to regional authorities together with financial resources (as encoded in the national budget law). Based on their autonomy, regional authorities organize the delivery of public health services. In the same way, environmental monitoring is carried out by ARPA, the regional authority for environmental protection.

Competences are shared between animal and human health within the same regional organization. Especially animal health control facilities operate in close synergy with the national system of the *Istituti Zooprofilattici sperimentali* (IZSs) on the Italian territory. IZSs operate under the mandate of the Ministry of Health. Hazard identification and quantification, as well as exposure assessment, characterize the surveillance activities performed by IZSs and ASL (public health and veterinary public health services) being aimed at designing the most effective sanitary measures to reduce negative health consequences on human and animals.

Universities are in charge of the training of public veterinarians and public health professionals; furthermore, their duty is to carry our researches in different fields connected to One Health, to provide scientific data and develop innovative solutions to mitigate the impact of biological (pathogens) and non-biological (pollutants) hazards on human, animal and ecosystem health. Research activities in this field are directly supported by EU Commission, the Ministry of University and Research, the Ministry of Agriculture and the Ministry of Environment. Universities may be indirectly supported by the Ministry of Health when performing researches with IZSs, ASL, ISS, IRCS etc.

University hospitals and Veterinary University Hospitals also play a key role on hazards surveillance sharing data and competences with local and national public health and the veterinary public health services.

Finally, the Ministry of Agriculture and Ministry of Environment share competences and strategies between them and the Ministry of Health. ARPA works at local/regional level in close cooperation with ASL and refers

to the Ministry of Environment, its duty is to identify and quantify potential environmental hazards that may directly or indirectly affect the health of human, animals and ecosystems.

Figure 3.1 - System description

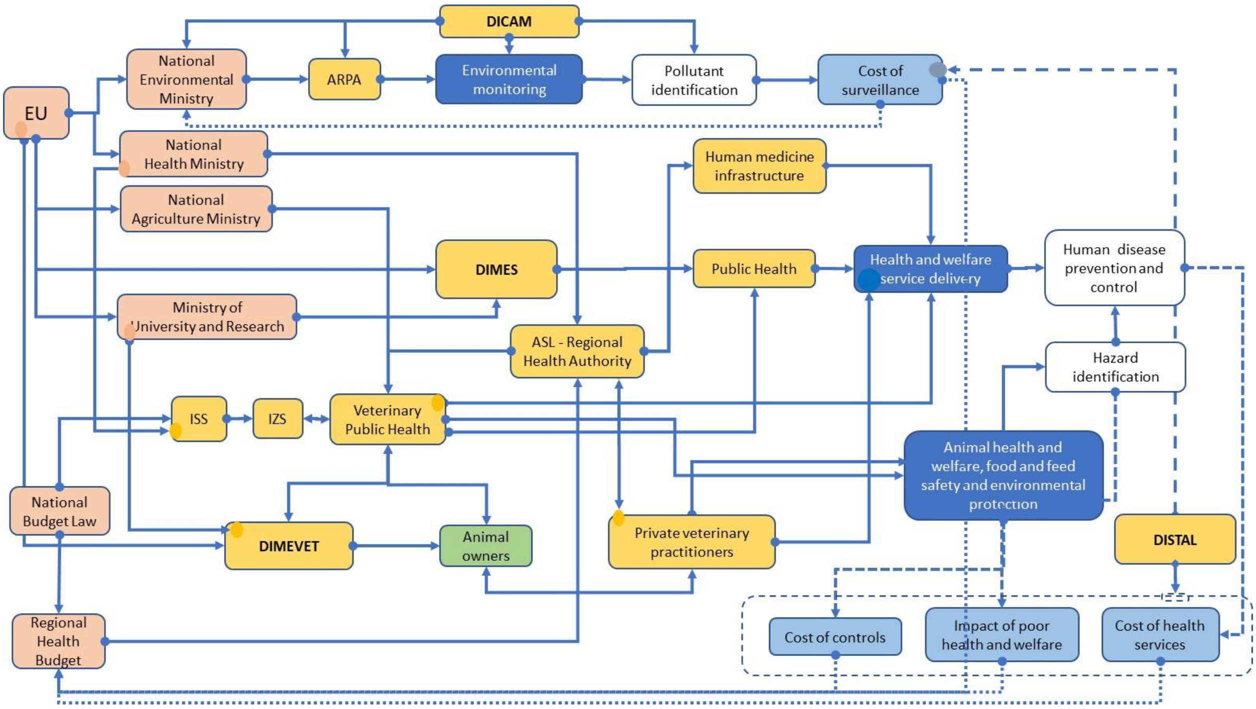

Decision-making processes concerning budget allocation (at both national and regional level) are poorly supported by economic information about the way animal diseases generate costs (the real dimension of costs, the way of cost transmission across the society, the economic link between animal health and animal health control, the cost of prevention and control of human diseases, food and feed control and environmental protection). Dashed lines connecting cost boxes with the regional health budget underline the economic problem and it's location.

Section 4: Theory of Change

In view of not complicating the diagram, here below a step-by-step explanation of the TOC.

INPUT: the research of the UNIBO team is based on international, national and regional public funders, and private funders, as well as UNIBO infrastructures.

RESEARCH: UNIBO team develops research activities in four main fields: epidemiology, economics, animal welfare and environment.

OUTPUTS: the outputs are the research results and are translated into science-based knowledge in the mentioned fields and into data on disease dynamics and determinants of health at the human, animal and environmental interface.

OUTCOMES: based on the acquired knowledge and the data obtained through research activities, the team expects the following outcomes: knowledge and technology transfer, dissemination among academic institutions and to society and the embedding of research findings into policy and communities. In addition, the team expects longer-term outcomes such as the reduction of human and animal disease burden and environmental contamination, the improvement of human and animal welfare, increased environmental preservation, the reduction of socio-economic and environmental impact of health hazards, enhanced preparedness, interoperability and prevention and increased awareness of and collaboration with local communities.

Figure 4.1 – Theory of change

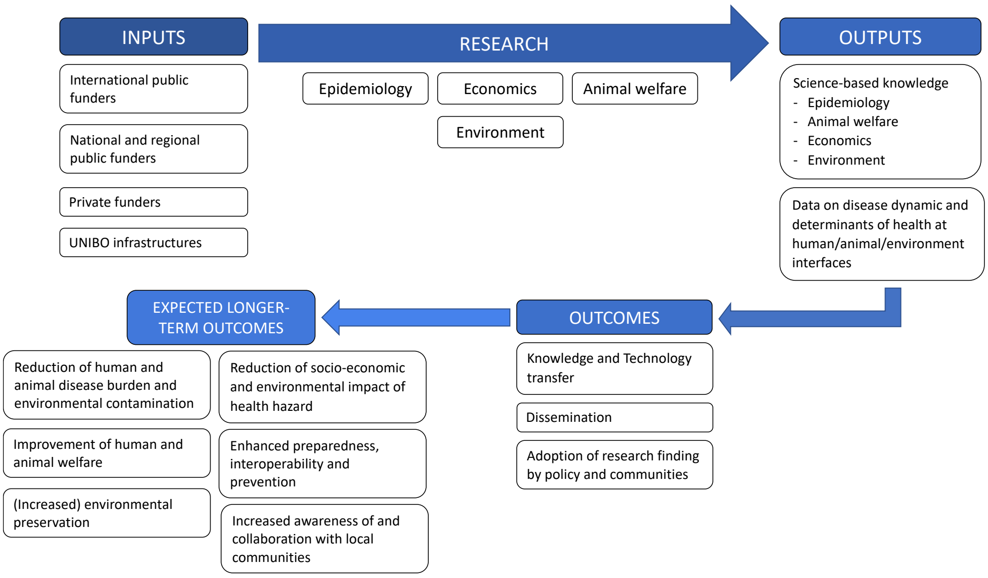

Section 5: Evaluation of OH-ness

As anticipated in the description of the team (Section 2, in particular points 10 and 12), the UNIBO team joined the first time in ELEPHANT. Before this experience, the members of the current ELEPHANT team used to work in almost separated groups belonging (and almost corresponding) to different scientific areas and/or departments (DIMEVET, DIMES, DICAM, DISTAL). More frequent relationship appeared among two of these departments (DIMES and DIMEVET), occasionally or on a systematic basis (e.g. sharing research or other academic activities), while the other 2 departments (DICAM and DISTAM) worked individually. All the early groups of the team actually implement the activity selected for the evaluation (research) in the same institution (University of Bologna), deal with OH for a relevant part of their research activity, have similar vision about the methodological innovation brought in by OH (need of inter-/trans-disciplinary approach and holistic or system approach, implementing them to some extent). On the other side, almost 2 groups did not share common research activities before their engagement in ELEPHANT, nor they worked or planned to work together. This situation may create difficulties at some stage of the evaluation, resulting in apparently contradictory scores. For example: the identification of the system and the definition of the TOC reflect the activity of all team members as far as all UNIBO researchers have common aims in relation to OH, even if they pursue them independently from each other and in different scientific domains. On the other side, at the moment of the evaluation some groups lack common facilities and protocols, they do not share specific research aims, projects or work routine. The situation of the UNIBO team can be described as in Figure 5.1.

Figure 5.1 – Structure of the team

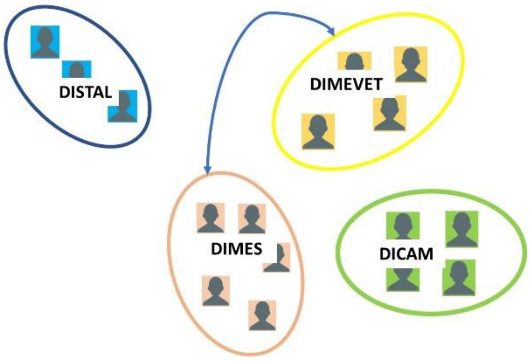

In few words, UNIBO team was not completely integrated before Elephant (or that it was partially integrated). The *degree of integration* can be assumed as a measure of the *structure of the team*. In Box 1 we propose a quantitative measure of the team structure trough the *integration index* (I).

Box 1 – Integration index of UNIBO Team

Integration Index $I = \Sigma C / \text{Max } C$

C = nr of relationships of each unit with the other units (see table below)
$\text{Max}_C = \Sigma U \times (\Sigma U - 1)$ = max nr of potential relationships within the team (for UNIBO team $\text{Max}_C = 12$)
U = nr of units (e.g. departments or other organization) taking part of the team (for UNIBO team $U = 12$)

$I_{\text{UNIBO}} = 0.167$

Units (U)	Contacts (C)	
DICAM	0	
DIMES	1	with DIMEVET
DIMEVET	1	with DIMES
DISTAL	0	
ΣU = 4	ΣC = 2	

Facing the above-mentioned situation, we asked ourselves how to score some attributes that could be influenced by the degree on integration of the team. This is a general problem of the evaluation in case of non-integrated teams, but affects in particular some aspects of Planning, Working, Sharing, Systemic organization. The solution we adopted (and that we propose) is to assign a score to each group within the team for the attribute or character to be evaluated, then calculate the average score (or weighted average) of the groups within the team and use this value as score of the team.[1]

5.1. Thinking

The element of Thinking is about the ability of the team to implement a system vision of the initiative in its context (system thinking). Evaluating Thinking is probably the most complicated task of the evaluation process. A relevant part of the preparatory work for this part of the evaluation was developed in Sections 2 to 4. In this part we build up on the information contained in those sections and go further by assessing the correspondence between system and initiative dimensions, the compliance of the initiative with general OH principles.

The questionnaire concerning this dimension is articulated in:

- 3 preliminary questions which aim at enumerating the main dimensions of the system and how they are considered in the initiative. The answers to these questions provide some basic data for the following evaluation questions.
- 11 evaluation questions which aim at evaluating how well the initiatives correspond to system thinking and comply with OH approach. The answers to these questions are used to score Thinking.

(See also Annexe B in *Conceptual framework OH-ness_Draft 08*). In the following, for each question we resume the reasoning behind the answer which justifies the score assigned.

---

[1] This problem may appear in different evaluation questions. For example, question 10 in Table 5.6 (Thinking) asks: *To what degree are the perspectives of stakeholders used explicitly to provide the backbone of the initiative?* This aspect may score differently by group A and group B. In this case the final score may result from the average of the 2 groups. Similar criteria can be adopted to take into account the structure of the team. Question 1 in Table 5.8 (Planning) asks about the existence of *Common aims in the initiative (was the initiative planned to reach a common overarching goal)*. Common aims do not exist if groups are not integrated, or they may exist among e.g. 3 groups out of 4. The score should take into account this situation weighting the score accordingly (e.g. by a weighted average which gives more importance to the score of the 3 groups).

The answers to some questions require a common reference framework to assign values, grades, intensity, etc. to dimensions or variables (for example: when we talk about the space or time dimension of the system, how can we grade the different levels of space and time? When we talk about life dimensions, how do we grade different levels of this dimension? This is a relevant aspect of the evaluation because the higher is the number of levels focused by the initiative, the higher is the score). Table 5.1 below anticipates such framework. We make explicit reference to the system dimensions adopted by UNIBO.

Table 5.1 – Reference framework for graduation

Dimension or variable	Levels or scales
Geographical dimension (space)	Local/regional<br>National<br>Multiregional/macro-region<br>International/EU<br>Planetary/worldwide
Time dimension	Very short/Contingency (from now to weeks)<br>Short period (weeks to months)<br>Medium term (few years)<br>Long term (many years)<br>Very long term (decades)
Dimensions of life	Cells and micro-organisms<br>Individuals (including animals, plants and humans)<br>Species (including animals, plants and humans)<br>Groups<br>Populations<br>Ecosystems<br>Environment (ecosystems + humans)<br>Planet/geosystem
OH sectors	Human health<br>Animal health<br>Plant health<br>Environmental health
Sustainability pillars	Society<br>Environment<br>Economy
Knowledge creation[2]	Basic knowledge, data<br>Trends, patterns and correlations<br>Models and complex relationships<br>Paradigm change, new vision
Teaching	First cycle (degree)<br>Second cycle (master)<br>Third cycle (PhD)<br>Life-Long Learning
Knowledge & technological transfer	Levels of this dimension can be graduated according to the degree of knowledge provided and the targeted audience<br>- Professional training for workers, to update their skills<br>- Technological transfer, specific knowledge transfer for innovation to the benefit of public and private institutions<br>- Dissemination, targeted to the wider audience
Social dimension	Individuals<br>Public institutions<br>Private institutions<br>Social groups (categories)<br>Grass-root movements

[2] Concerning the levels of this dimension we can assume the partition suggested for Q4 in Thinking sheet, column *Brief element description*. Knowledge creation can be aimed at simple data collection and basic statistical elaboration (descriptive), at the identification of patterns (trend identification, correlations), model construction (understanding complex relationships and links among factors) or changing the way of thinking and the assumptions behind the scientific activity (change of perspective or paradigm).

	Society as a whole
Economy	Individuals (firm, farm, consumer)<br>Markets<br>Sectors<br>Supply chains<br>Society<br>Finance
Features of an initiative	An initiative can be thought, designed and planned in order to:<br>- target individual events<br>- modify trends or patterns of a phenomenon<br>- modify the factors which make the system work

a. Preliminary questions

Question P1: *How strongly would the context of the initiative (system) be affected, if the dimension was excluded?*

The answer is based on the description of the system (see Section 3) having in mind, at the same time, the initiative as described in Section 2 (in particular questions 5 to 7). We then developed a reasoning to justify the relevance of each dimension (during this process other relevant dimensions may appear or deemed relevant; if so, add those dimensions here and amend the system figure consequently).

The scoring assigned to each dimension (from 0.0. to 1.0) should reflect the importance of the dimension in the system (0.0 = no importance; 1.0: highest importance).

UNIBO team identified 8 dimensions of the system. Such dimensions are listed in the table below with a brief reasoning supporting either the choice and the scoring of its importance (in brackets).

Table 5.2 – System dimensions

**Dimensions**	**Supporting reasoning**	Score
Geographical dimension	The system we designed involves different functional spaces, from regional/local to national, where the activities mentioned in the system map take places. Further, the space is a relevant dimension of several research activities in the system (epidemics, hazard identification, environment, economy, etc.: in all of this issues space holds a key function). Further, the system is not isolated from the rest of the world, the way it works does have spatial dimension crossing the places where phenomena mentioned in the system occur (epidemics, environment). This said, we should ask ourselves how much system functioning would be affected if we eliminate this dimension: is this dimension crucial or it has a minor/non vital importance? Space is important but is not the core of the system, but its relevance should be considered very relevant especially in view of the recent pandemic events.	0.8
Time	Almost the same reasoning as for space: the timeline of diseases effects, of economic effects, are relevant but are not at the core of the system.	0.4
Dimensions of life	Dimensions of life are very relevant for the system as they are the core of the health consequences occurring in the system at different levels (environment, animals, human beings, populations).	1.0
Knowledge creation	This dimension (actually, research) is a core dimension of the system as it provides the scientific means to intervene on health at different levels (environment, animals human ecc.)	1.0
Teaching	Teaching is a crucial dimension of the system to transmit scientific knowledge and competences to next generation, and professionals including Long Life Learning.	0.8
Knowledge & technological transfer	Knowledge & technological transfer are the natural outcomes of knowledge creation and allow the society to get benefits from it in terms of effectiveness and efficiency in all sectors related to health across time and space	0.8

Economy	Though not a priority in the system, economy is relevant to understand the economic consequences of diseases and provide data for a proper use of the scarce resources devoted to health in any sector	0.6
Social dimension	Public and private institutions and stakeholders are relevant actors of the system. Public institutions provide rules and policies which influences system functioning, individual and social behaviours, resource allocation in the respect of health at any level; stakeholders benefit of knowledge creation and knowledge & technological transfer to the benefit of the whole society, playing a relevant role in the improvement of health and safety and can co-operate with public institutions to strengthen effectiveness.	0.8

Question P2: *What importance is given to the different dimensions within the initiative?*

This question aims at assessing the importance of each dimension within the theory of change and the plan of the initiative. The answer should be based on the TOC figure (see above Section 4 and Figure 4.1) and evaluate how much/how well each dimension is considered in view of its expected effect, according to the TOC.

Scoring: 0 =not considered, 0.2 = passive recognition. 0.4 =considered, 0.6 =weak involvement, 0.8 =strong involvement, 1= essential

Table 5.3 – Importance given to system dimension in the initiative

Dimensions	Supporting reasoning	Score
Geographical dimension	The geographical dimension of the research developed in the initiative crosses in some cases geopolitical and geographic borders, as also witnessed by the input structure (national and international funds). Despite the local character of some activities (e.g. research and outputs may have local dimension), outcomes and impacts may have greater spatial importance (e.g. may be used in other geographical context). Finally, the geographical dimension is implicit in the study of disease dynamic at different interfaces (human, animal, ecosystems and environment)	0.8
Time	Time plays a relevant role in the TOC as research needs time (studies last 2 to 4 years), and the process from activities to outcomes can take years too. The activity does not pay particular relevance to this problem (e.g. to speed up research and get result, or to reduce the time to implement result)	0.2
Dimensions of life	Dimensions of life are crucial in the TOC as outcomes and impacts strongly depends on the output related to the interaction among different life dimensions	0.8
Knowledge creation	This dimension is crucial in the TOC (key output) and the basis to start further steps of the TOC. This dimension is given specific importance and resources in the TOC	1.0
Teaching	Teaching is well considered in the TOC as research result are translated in teaching contents and methods	0.8
Knowledge & technological transfer	As above.	0.8
Economy	As above, but it actually plays a secondary role in the TOC as economic research is limited to few aspects of the TOC.	0.4
Social dimension	Most of research outputs (especially on the medical side, both human and animal) directly affect the social dimension (e.g. human and animal health care)	0.6

Question P3: *How many scales are considered in the different dimensions of the initiative?*

This question aims at assessing the articulation of levels within a dimension. Each dimension can include different levels or scales (e.g. for the geographical dimension, the scale considered by the initiative can be a region, the state, or an international dimension, or all of them. See Table 5.1 for reference).

The scoring enumerates the number of scales of each dimension (e.g. if the initiatives takes into account the regional and national scale, the score for this dimension is 2)

Table 5.4 – Scales of the dimensions

**Dimensions**	**Scales**	**Score**
Geographical dimension	1. Local/regional<br>2. National<br>3. International	3
Time	1. Very short/Contingency (from now to weeks)<br>2. Short period (weeks to months)<br>3. Medium term (few years)<br>4. Long term (many years)	4
Dimensions of life	1. Cells and micro-organisms<br>2. Individuals (including animals, plants and humans)<br>3. Species (including animals, plants and humans)<br>4. Groups<br>5. Populations<br>6. Ecosystems<br>7. Environment (ecosystems + humans)	7
Knowledge creation	1. Data<br>2. Trend/patterns<br>3. Models<br>4. Paradigms	4
Teaching	1. First cycle<br>2. Second cycle<br>3. Third cycle<br>4. Life-Long Learning	4
Knowledge & technological transfer	1. Training for professionals<br>2. Technological transfer<br>3. Dissemination to wider public	3
Economy	1. Individuals (firm, farm, consumer)<br>2. Markets<br>3. Sectors<br>4. Supply chains<br>5. Society<br>6. Finance	4
Social dimension	1. Individuals<br>2. Public institutions<br>3. Private institutions<br>4. Social groups (categories)<br>5. Grass-root movements<br>6. Society as a whole	6

b. Scoring questions

Scoring questions are articulated as in Table 5.5. below. They aim at evaluating the level of system thinking adopted in the initiative and the correspondence of the initiative with the basic OH characteristics

Table 5.5- Attributes of Thinking

System dimensions considered by the initiative and their balance	Question 1
Correspondence between initiative's dimensions and the dimensions of the context	Question 2
System features	Question 3-5
TOC-factors	Questions 6-8

OH-specific factors considered	Questions 9
Social perspectives	Questions 10-11

The final Thinking score results from the average of the scores of each question. Scoring criteria are different for each question. In Table 5.6 below we report the questions and their meaning, the supporting reasoning when relevant, the scoring criteria and the scores.

Table 5.6 – Scoring questions: Thinking

Question	Focus of the question	Supporting reasoning	Score
System dimensions considered by the initiative and their balance			
1. How balanced is the consideration of the different dimensions by the initiative?	This question assesses whether the initiative has a balanced focus on the dimensions that are considered important in the initiative and in the theory of change.<br>Scoring combines the number of dimensions and the number of levels of each dimension (see answer to question P2 (***See note 2***)<br>(Score=1-[(number of different scores-1)/number of dimensions].	We identified 8 relevant dimensions of the system (see answer to question P1) and assigned 4 different scores in answer to question P2.	0.50
Correspondence between initiative's dimensions and the dimensions of the context			
2. How well does the consideration of dimensions by the initiative match the dimensions of the system in which it operates (context)?	Assessing the match between the initiative and the system (context) within which it operates.<br><br>Scoring:<br>0 =no match / 0.2=very weak match / 0.4= weak match / 0.6 = good match / 0.8 =strong match/ 1 = perfect match	The way dimensions of life, knowledge creation, teaching and technological transfer are considered in the initiative have a strong correspondence with the dimensions of the systems. Actually research (Knowledge creation) is the core activity of the team, naturally ending in teaching, dissemination and technological transfer. Social dimension matches well but could probably have more relevance in the initiative. Economy, space and time dimensions of the initiative are addressed less well than their relevance in the system would require<br>Based on the answers to questions 1 and 2, the match between system and initiative dimensions are: Space: 100%; Time: 50%; Dimension of life: 80% - Knowledge creation: 100/;	0.9

		Teaching: 100%; Knowledge and technological transfer: 100% - Economy: 67%. Social dimension: 75%. The score is around 0.9	
System features			
3. How well does the number of dimensions and scales reflect an integrated approach?	This question assesses whether the number of dimensions and the scales within those, which are considered in the approach, are sufficient to consider the initiative an integrated approach.<br><br>Scoring:<br>0= 1 dimension, 1 scale<br>0.2=2-3 dimensions, 1 scale<br>0.4=1 dimension, >1 scales<br>0.6=2-3 dimensions, of which one >1 scales<br>0.8=2-3 dimensions, few at > 1 scales<br>1= very comprehensive	Scoring stems from considering answers to questions 1 and 3, and it is graded according to the combination of the number of dimensions and the number of scales. (***see note 3***)<br><br>We identified 8 system dimensions, most of them having 4 dimensions considered by the initiative	1.0
4. To what degree does the initiative identify subsystems and interactions among them and integrate this structure in the theory of change?	Assessing to what extent the structure of the system and the hierarchies within system components have been considered<br><br>Scoring: qualitative appraisal translated in a scale:<br>0 = no consideration<br>0.2 = essential subsystems described<br>0.4 = some scales described<br>0.6 = some scales and some sub-systems embedded<br>0.8 = many subsystems and scales embedded<br>1 = essential sub-systems and scales embedded	Considering the different activities of the team, different sub-systems are considered (human health, animal health, economy, environment) but, these sub-systems partially act in coordination, some of them act almost independently with scarce team integration (e.g. economists are not integrated with health and environment issues treated by the other parts of the team, more integrated with each other). This lack of coordination also reflects in the integration of activities in the TOC and its implementation	0.6
5. How are the dynamic feedback loops of the system identified?	This question assesses to what extent the complexity of the relationship among system units is identified<br><br>Scoring: Qualitative appraisal translated in a scale from:<br>0= no loops identified<br>0.5 = essential loops identified<br>1= detailed causal loop analysis	The system figure outlines complex relationships among several units of the system, with different functions (political and administrative, scientific, operational) and space dimensions. Some dynamic loops and feedback are considered, including those involving the environment,	0.6

		the animal and human health.	
TOC factors			
6. What feature (level, event) of the system is targeted by the initiative?	This question aims at assessing the complexity, comprehensiveness of the level targeted by the initiative. An initiative can be thought, designed and planned in order to: (i) target individual events; (ii) modify trends or patterns of a phenomenon; (iii) modify the factors which make the system work (see Table 5.1) (***see note 4***).<br><br>Scoring:<br>0.2=events<br>0.6=patterns<br>1=structures	Most of activities are targeted at events and patterns. Some are targeted at modifying structures (ROADMAP, ….)	0.8
7. Are time delays recognised in the theory of change?	Assessing the timing of the processes activated by the initiative. This aspect is crucial for the organization of the initiative and its effectiveness. This aspect should be well defined in the TOC where the distinction of outputs, outcomes and impact has a causal and time definition (what-determines-what necessarily implies a timing)<br><br>Scoring:<br>Score the consideration of delays on a scale between 0 (no consideration) and 1 (detailed time analysis).	Time delays are considered as a relevant aspect of the initiative by many of the research domains and do have consideration in the TOC (e.g. epidemiology, molecular epidemiology).	0.4
8. Where is the initiative situated in relation to the chain of events causing the problem, and responding to it?	Assessing what kind of effect is expected on the problem. This question complements question 4<br><br>Scoring:<br>0= correcting damage<br>0.2= containing damage<br>0.4= preventing damage<br>0.6= modifying behaviours<br>0.8= modifying structures and paradigms<br>1= modifying the socio-ecological system	The initiative targets several aspects of the system: knowledge creation with research, teaching, knowledge and technological transfer, several dimensions of life, economic evaluation. Altogether, the activity involves several key aspects of the system. Considering the TOC, the expected result is aimed at improving the health and welfare animals and ecosystems by preventing diseases and disorders, adverse situations and behaviours, with an eye to the modification of behaviours and structures	0.8

OH-specific factors considered			
9. How well does the initiative consider One Health and the three pillars of sustainability?	Assessing the intrinsic OH aspects of the initiative. One Health integrates the aspects of human, animal, plant and environmental health. Sustainability relies on the three pillars of society, environment and economy (see Table 5.1)<br><br>Scoring:<br><br>0=single pillar and aspect<br>0.2=one aspect 2 pillars<br>0.4=one aspect 3 pillars<br>0.6=two aspects 2 pillars<br>0.8=two aspects 3 pillars or 3 aspects 2 pillars<br>1=integrates 3 and more of each	Pillars: society, economy and environment are considered in the activity (3 pillars). Animals, humans and the environment are also considered (3 OH aspect out of 4)	0.8
Social perspectives			
10. To what degree are the perspectives of stakeholders used explicitly to provide the backbone of the initiative?	Assessing to what extent the different ethical, epistemological, and methodological grounding of different stakeholders have been considered in the initiative<br><br>Scoring: qualitative synthetic appraisal translated in a scale from 0.0 (no consideration) to 1.0 (complete consideration)	Some stakeholders are involved, but there is not a systematic and structural stakeholder involvement in the initiative.	0.6
11. To what degree does the initiative consider the beliefs about evidence, values about health, cultural grounding as factors affecting the theory of change?	Scoring: Qualitative synthetic appraisal translated in a scale from 0.0 (no consideration) to 1.0 (complete consideration)	The initiative has a poor consideration of cultural factors	0.2

Notes to Table 5.6

1. In the process for the selection of the relevant dimensions the following two questions should be raised: 1) Is this dimension important for the initiative, or can it be excluded without effect on the expected and unexpected outcomes of the initiative? 2) Does the initiative have an effect on the dimension?
2. Assume that we have 5 dimensions in the initiative (e.g. space, time, economy, organization and regulation) which, according to answer to question pp2, respectively scored: space= 0.8; time= 0.8; economy= 0.6; organization= 0.5; regulation= 0.5. The number of different scores is 3 (0.8, 0.6 and 0.5).
3. Scoring is assigned according to the following combination of dimensions and scales:

0 = 1 dimension, 1 scale	0.2 = 2-3 dimensions , 1 scale	0.4 = 1 dimensions, > 1 scale
0.6 = 2-3 dimensions, of which one > 1 scale	0.8 = 2-3 dimensions, few at > 1 scale	1= very comprehensive

Making reference to the example of footnote 14, if 5 dimensions are considered according to the following scales: space= 2 scales (regional, national); time= 1 scale (years); economy= 2 scales (firm, sector); organization= 3 scales (cooperative of firms, national public organization, international agencies); regulation= 3 scales (regional, national, EU), the score would 1.

The COVID-19 case: if the contingent problem is the high rate of infection and related shortage of places in hospitals, the objectives considered by the initiative may be: (i) build-up camp hospital to host the increasing number of patients (single event targeted in the short run); (ii) promote measures to limit contagion (social distance, isolations, movement restrictions; result expected in the change in infection trend); (iii) increase early detection mechanism, define international protocols, increase structural flexibility of care facilities (structural change of the system, preparedness for future scenarios; result expected in the long run). The assumption behind scoring is that the higher is the level of the intervention (more general, more comprehensive) the more it complies with an integrated, OH-vision of the problem.

c. Scores and comments

Balance of system dimensions	0.5
Initiative vs. System	0.8
System features	0.8
TOC factors	0.6
OH issues	0.8
Social perspectives	0.4
**Overall Thinking score**	**0.60**

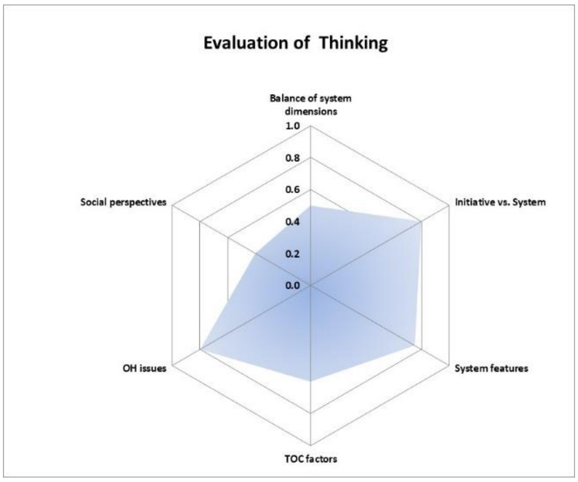

5.2. Planning

Planning refers to how the aims foreseen by an initiative are implemented in practice. Considering the system vision of the initiative (the way we perceive and understand the initiative in the context) and the theory of change (the way we foreseen actions make result), planning translates ideas into programmed actions. Actually, planning is a process which requires that resources are allocated to specific actions to obtain result, and that responsibilities are assigned consequently based on competences. Some aspects should be carefully considered in a OH perspective. Generally speaking, preliminary conditions of planning require that resources are adequate to the objectives and roles and responsibilities reflect competencies. More specifically, planning according to OH concept should provide the organizational means to make system vision and inter-/trans-disciplinarity effective and dynamic (i.e. flexible and adaptable to change), crossing disciplinary,

organizational and institutional borders as much as possible.[3] Secondly, initiative's objectives should reflect as much as possible a common understanding of the team, including not only actors (e.g. team members) but also stakeholders. Finally, partial achievements should be regularly assessed to adapt planning and organization to change.

a. Scoring questions

The evaluation is based on 8 scoring questions structured in 4 main sections reflecting the desirable attributes of Planning listed in Table 5.7

Table 5.7 – Attributes of Planning

Common aims	1-2
Stakeholder and actor engagement	3-5
Self-assessment and plan revisions	6-7
Objective(s)	8

Table 5.8 – Scoring questions: Planning

Question	Focus of the question	Supporting reasoning	Score
Common aims			
1. Common aim(s) in initiative	The question tries to assess if the initiative was planned to aim for a common overarching cross-sectoral/-disciplinary goal relevant to One Health including health, societal, environmental and/ or economic outcomes (sustainable solutions)<br>Scoring:<br>0=no common OH-relevant aim(s)<br>0.2=few/little common OH-relevant aim(s)<br>0.4=some common OH-relevant aim(s)<br>0.6=common and OH relevant aim(s)<br>0.8=highly common and OH relevant aim(s)<br>1=clear common and fully OH relevant aim(s)	Despite the convergence toward common OH aims among the sub-groups, at the time of the evaluation they developed their activity almost independently from each other without a common decision about aims. Given the structure of the team (see Section 2 points 10 and 12; and Box 1 in Section 5), there was almost no common aim in the initiative except for DIMEVET and DIMES	0.3
2. Planned organisation needed to reach common aim(s)?	Is/was the planned organisation in terms of staff positions and involvement, participating institutions, networks, communication pathways, leadership etc. of the initiative relevant and needed to achieve the stated aim(s) (i.e. is a One Health approach supported?)<br>Scoring:	There was no planned organization if we consider the initiative as a whole (as mentioned above, individual groups were at work almost independently). On the other side, the general environment and	0.3

[3] Sectoral or disciplinary aims codified in rigid institutional mission, rules and organization may rise walls against inter and trans-disciplinarity which may reflect in the impossibility to plan OH activities (e.g. financing rules of an institutions may hinder resource delivery for objectives which are not codified by the institution itself). This may happen in scientific as well as in administrative institutions. Planning according to OH needs that such barriers are transcended.

	0=organisation not supportive of OH approach<br>0.2=organisation slightly supportive of OH approach<br>0.4=organisation somewhat supportive of OH approach<br>0.6=organisation supportive of OH approach<br>0.8=organisation highly supportive of OH approach<br>1=organisation fully supportive of OH approach	facilities of the main institution (Unibo) play a role in facilitating the convergence of individual groups toward a common aim (info circulation, institutional routines, etc.). Institutional resources played a general role in supporting individual groups activities and some networking.	
Stakeholder and actor engagement			
3. Actor identification process ('the right people heard?)	Has a process to identify and engage all essential stakeholders (e.g. governmental, academia, industry, NGOs, general population, etc.) been used in the planning of the initiative?<br><br>Scoring:<br>0=no identification process<br>0.2= minor identification process<br>0.4= identification process partly used<br>0.6= reasonable identification process used<br>0.8= highly relevant identification process used<br>1= perfect identification process used	There was not a planned, shared process to identify stakeholders. Stakeholders involvement occurs ex facto (e.g. because of financial support) but not according to a planned activity in the teams or sub-groups	0.3
4. Actor identification process ('the right people involved'?)	Has a process to identify and involve essential actors (including governmental, industry, health units, professionals, technicians, etc.) been described and followed in the planning of the initiative? Do/did the initiative consider the influence of the experiential history of the actors on the understanding of the system? Are/were motivations and aversions of actors explained based on their history in the context (system)?<br>Scoring:<br>0=no identification process<br>0.2= minor identification process<br>0.4= identification process partly used<br>0.6= reasonable identification process used<br>0.8= highly relevant identification process used<br>1= perfect identification process used	As above	0.3
5. Planning of engagement of stakeholders, use of stakeholder input and effect	How is/was the stakeholder engagement planned to ensure a One Health approach in the working and organisation of the initiative aiming to achieve the stated objectives and One Health outcomes? Does/did the initiative	As above	0.2

on stakeholder perceptions	contain a mechanism to change methods or gage involvement of new actors, based on stakeholder analysis or feedback? How does/did the knowledge of stakeholders feed back into the governance of the initiative? How clear is it outlined how the stakeholders might converge towards a common understanding of the system?<br><br>Scoring:<br>0=no stakeholder engagement, no feedback<br>0.2=little stakeholder engagement, e.g. questionnaires<br>0.4=some stakeholder engagement, e.g. workshops<br>0.6=reasonable stakeholder engagement, e.g. participation in implementation<br>0.8=high level of stakeholder engagement, e.g. participation in planning<br>1=very strong stakeholder engagement, e.g. participation in planning and adjustments throughout initiative		
Self-assessment and plan revisions			
6. Is the initiative built on an iterative process?	Does the initiative allow for 'underway'-corrections in planned activities or organisation in relation to its theory of change? To what degree does the initiative revisit, assess and revise decisions and opinions iteratively, and are these plans reasonable in relation to the time dimensions in the system and theory of change?<br>Scoring:<br>Score the iteration frequency on a scale between 0 (no reassessments) and 1 (very frequent reassessment/reassessment sensibly distributed in relation to the relevant time dimensions).	There is not a structured reiterative process to critically revise the activity at regular point in time. This happens anyway through routine practices leading to result assessment and activity readdressing	0.5
7. How appropriate is the time and budget allocated for self-assessment?	Score the adequacy of the time and budget<br><br>Scoring:<br>Score the adequacy of the time and budget on a scale between 0 (no time or budget) and 1 (adequate).	There is not a monetary budget for reassessment but time allocation for this task is relevant and crucial for the activity to be effective. Time is regularly allocated.	0.5
Planning of specific objectives:			
8. Planning of resource allocation for specific OH objectives (more objectives of the	How well did the planning of tasks and resources to fulfil these tasks match for this objective of the One Health initiative? Did this planning and resource allocation underpin achievement of the objective and hence anticipated OH-outcomes?	Specific objectives are pursued by almost all the groups, individually or in partial integration. Most researches are financed by public or private funders and	0.7

initiative can be considered)	Scoring:<br>0 = planning and resources not supportive<br>0.2 = planning and resources slightly supportive<br>0.4 = planning and resources somewhat supportive<br>0.6 = planning and resources supportive<br>0.8 = planning and resources highly supportive<br>1 = planning and resources fully supportive<br><br>Additional notes about scoring:<br>- List and score the main projects developed by each thematic group(s) according to Section 2, point 1.<br>- Calculate the mean for each thematic group<br>- Calculate the general average of all the groups	budget is properly allocated for the aims. A relevant part of the research falls within OH domains. In the next lines we consider and score resource planning for the specific objectives by thematic area as identified in Section 2, point 1. In the evaluation we consider the TOC, in particular Outputs and Outcomes	
8.1. Objective 1	Economics	ROADMAP	1.0
		BLUETONGUE	1.0
		BRC	1.0
		ECHIN.	0.2
		**Average**	**0.8**
8.2. Objective 2	Veterinary area	VETBOME (2018/2019), Formation	0.5
		LAMP (2016/2019)	0.6
		Diagnostic methods viral diseases in bovines	0.7
		Streptococcus agalactiae	0.8
		AMR reduction	0.5
		FILAVAL	0.5
8.3.		**Average**	**0.6**

b. Scores and comments

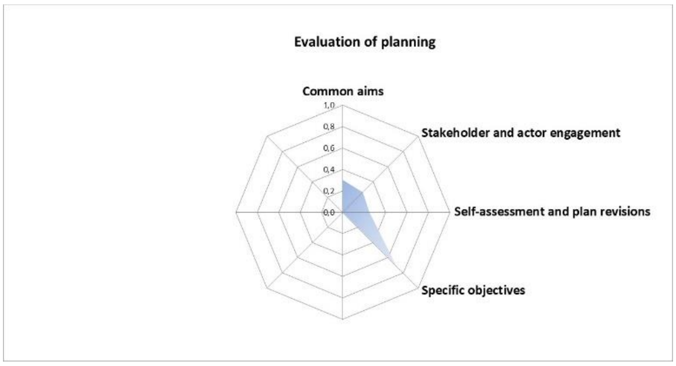

5.3. Working

Working focuses on the usual practices and work routines developed inside the team which lead to the interdisciplinary and participatory (i.e. transdisciplinary) engagement of the team in the initiative. Leadership and management are key factors to implement trans-disciplinarity through strategic dialogue and shared decision-making, which in turn foster a non-hierarchical relationship between the different disciplines and members within the team. Working methods should also allow for self-reflection, flexibility and recursiveness, so that underlying assumptions and concepts can be challenged and modified as learning leads to an enriched understanding (Rüegg, 2018).

The questionnaire is articulate in:

- 1 preliminary question concerning the relevance of inter-/trans-disciplinarity in the initiative
- 10 scoring questions concerning the desirable attributes of the working routine in the team

a. Preliminary question

Question 1: *Is inter-/trans-disciplinarity required to solve this problem? And what are the benefits of using a transdisciplinary approach in the initiative rather than conventional/disciplinary approaches?*

Score the need for trans-disciplinarity on a scale between 0=not required and 1=cannot be achieved without. (This score is not counted into the overall scoring system.

The answer wants to assess how relevant is a inter/trans-disciplinary approach in the initiative. As mentioned in Section 2 (in particular points 1 and 3: diversity of scientific domains and the need to strengthen interdisciplinary links; point 2: need to affirm the holistic vision of problems), inter- and trans-disciplinarity are strongly needed in the initiative. Benefits of such approaches lie in the possibility to increase the knowledge of complex problems and to gain effectiveness and efficiency in the use of the resources.

Scoring: 0.8

b. Scoring questions

Table 5.9 – Attributes of Working

Broadness of initiative	1-3
Integration and collaboration	4-5
Transdisciplinary balance	6.1-6.3
Cultural and social balance	6.4-6.7
Flexibility and adaptation	7-10

Table 5.10 - Scoring questions: Working

Question	Focus of the question	Supporting reasoning	Score
**Broadness of initiative**			
1. How diverse are/were the disciplines, methods, scales of analysis and/or social actors involved?	Score the diversity on a scale between 0 and 1, with 0 = very homogeneous project (e.g. only natural science, no interdisciplinarity, no participation: laboratory experiment), 0.5 intermediate diversity (e.g. two disciplines or two sectors, primarily natural or primarily	Data to answer this question can be retrieved from previous questionnaires:<br>High number of different disciplines	0.8

Enumerate all disciplines, methods, dimensions and scales of analysis considered, as well as the social actors involved, as they were introduced in the OH Thinking.	social science, government agency and academia: disease surveillance in a food chain), 1 high diversity (e.g. participatory epidemiology, qualitative and quantitative approaches in natural and social science, integrated approach to develop a locally sustainable food system)	takes part in the team. At least 6 distinguished disciplines or thematic areas: veterinary medicine, human medicine, epidemiology, animal production, environmental science, economics (see Section 2)<br>Several features of the system considered (see Table 5.6-Thinking, answer 6); quantitative and qualitative approaches, aimed at having effect up to structural change (idem, question 8); 3 out of 4 OH sectors considered (idem, question 9)<br>The diversity of disciplines, methods etc. is thus high or very high	
2. To what extent is/was the non-scientific community involved during the execution of the initiative?	This question aims at assessing the degree of interaction among disciplines (multi and inter-disciplinarity) and among institutions beyond academia (trans-disciplinarity).<br><br>Scoring:<br>No, score = 0; community is consulted for problem definition, score 0.3; community is also consulted to develop solutions, score 0.6; community contributes to monitoring, implementation and/or decision-making, score =1.	As for Economics, the degree of involvement of inter/transdisciplinarity varies from 2 (national projects) to 4 or 5 disciplines in EU projects (ROADMAP). The same difference holds for stakeholders involvement. In average the score could be 0.5	0.5
3. To what extent is/was cross-sectorial involvement present during the execution of the initiative?	This question aims to identify whether the initiative involves and crosses different sectors of the society. Sectors should be understood in this case as separated activities of economy and society showing some intrinsic homogeneity, e.g. human health, human health, production, education, research, etc. (this closely recall the definition of sector adopted in Economics)<br><br>Scoring<br>One sector involved, score 0; two sectors involved, score 0.3; three sectors involved, score 0.9; more sectors involved, score 1.	The activities developed by the team (also considering the structure of the team i.e. the individual groups) usually involve different sectors: human and animal medicine, animal production, environment, economy. Cross-sectorial involvement should rank moderately high.	0.7

Integration and collaboration			
4. To what extent do/did the different disciplines work together?	The question aims at assessing the experience and the practice of interdisciplinary/ transdisciplinary working of the team, which can be assessed by the frequency of meeting of the team, by the degree of participation in all team activities.<br><br>Scoring: reflect on the questions below and score consequently.<br>(i) Are meetings with all disciplines (face-to-face or virtual) held frequently?<br>(ii) Are aims and objectives shared and clear to all?<br>(iii) Is there joint decision-making?<br>Score: 0= none of the above; 0.3=one of the above; 0.6=two of the above; 1=all of the above	Each sub-group used to meet frequently but the number of disciplines involved was limited (2-3) due to the structure of the team at the moment of the evaluation (see Box 1)	0.6
5. How is interaction between people organised to foster collaboration across the initiative?	This question aims at assessing what means, devices or routine are in place to promote and maintain the collaboration among team members. The following issues should be considered: are there face-to-face interactions across the initiative? If yes, how frequent are these interactions? If not, what is done to overcome potential misunderstandings, lack of information and collaboration?<br><br>Scoring:<br>Score the level of interaction to encourage collaboration on a scale between 0 and 1, with for example no interaction/no collaboration, score =0; little or rare interaction, score = 0.2; quarterly meetings/Skype meetings, score 0.6; monthly planned in-person meetings, score 0.8; spontaneous frequent in-person meetings, score 1.	Material and virtual meeting are regularly held among team members. These meeting do not involve all the team members due to the team structure at the moment of the evaluation (see Box 1). This said, in general meetings (physical and virtual ones) are regularly held 2 to 4 times per month	0.6
6. Are/were there power (i.e. academic or disciplinary dominance) or gender imbalances within the initiative, which risk biasing the process?	This question probes for the dominance of one discipline over the rest. The answer is articulate in 7 sub-questions to assess the balances in different situations, which can be classified as *trans-disciplinary* and *cultural-social* balance<br>Scoring<br>Power clustering, uneven power distribution, for each score 0; strong empowerment of all participants, score =1.		
Trans-disciplinary balance			
6.1) Across disciplines		Natural sciences are predominant in number but this does not	0.8

		generate power imbalances	
6.2) Across sectors		Health sectors are more represented (animal and human medicine), followed by environmental and economic sector	0.8
Social-cultural balance			
6.3) Across ethnicities	Is/was there an imbalance in ethnicities of participants?	Are different ethnicities present? Are they discriminated?	0.0
6.4) Across social classes	Is/was there an imbalance in social classes involved?		1.0
6.5) Across gender	Is/was there an imbalance in gender of participants?	The team is made of 5 female and 5 males	1.0
6.7) Cultural issues	This question looks for contexts which may inhibit the success (issues may include, mobbing, unequal task distribution based on traditions, paternalistic behaviour, war, instability, poverty etc.)		1.0
6.7) Religious issues	This question looks for religious issues which may inhibit the success		1.0
Flexibility and adaptation			
7. How likely is reflection going to feed back into corrective action within the initiative?	The question assesses whether the initiative will connect reflection and action.<br><br>Scoring<br>Score the likelihood on a scale from 0 to 1 with no feedback, score =0, feedback without action, score =0.3, feedback and defined action pathway, score =1.	Reflection is part of the discussions inside the group to check result, take into account criticisms and adjust. There is no a regular protocol to perform this process	0.7
8. How flexible is the initiative design and timeline to respond to internal or external changes at short-term?	The question checks for feasibility of project design and timeline in the short-term.<br><br>Scoring<br>Score the flexibility on a scale from 0 to 1, with 0 no flexibility, 1 high flexibility	Flexibility depends much on rules of engagement with funders and sponsors of the initiative. It's likely to take place at the beginning of the activities but lowers as the activities are implemented	0.7
9. How flexible is the project design and timeline to respond to internal or external changes at mid-term?	The question checks for feasibility of project design and timeline in the medium term.<br><br>Scoring<br>Score the flexibility on a scale from 0 to 1, with 0 no flexibility, 1 high flexibility	Low flexibility is possible from mid-term ahead	0.6
10. How flexible is the project design and timeline to respond to internal or	The question checks for feasibility of project design and timeline in the long-term.<br><br>Scoring	Almost no flexibility in the long term	0.3

external changes at long-term?	Score the flexibility on a scale from 0 to 1, with 0 no flexibility, 1 high flexibility		

c. Scoring and comments

Broadness of initiative	0.7
Collaboration	0.6
Transdisciplinary balance	0.8
Cultural and social balance	0.8
Flexibility and adaptation	0.6
**Overall Working scoring**	**0.7**

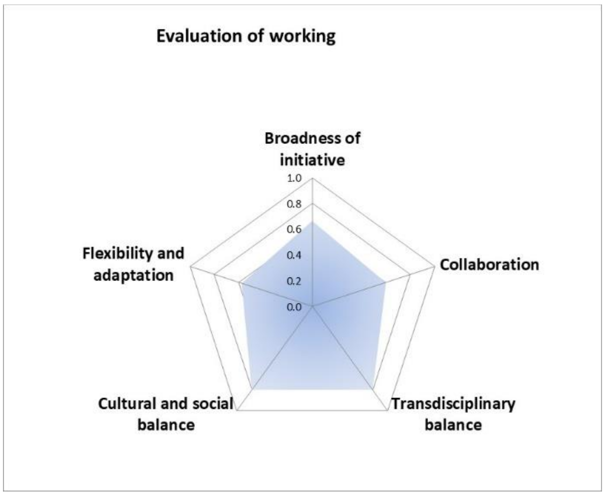

5.4. Sharing

Sharing dimension basically deals with protocols and facilities adopted by the team to ease storing and access to information, methods and result of the activity. Sharing methods should be effective both inside and outside the team (e.g. toward the relevant organizations and the environment).

a. Scoring questions

Table 5.11 – Attributes of Scoring

General information/awareness sharing	1-3
Data and information sharing	4-6
Methods and results sharing	7-8
Institutional memory/resilience	9-10

Table 5.12 – Scoring questions: Sharing

Questions	Focus of the question	Supporting reasoning	Score
General information/awareness sharing			

1. Internal mechanisms for sharing general information/awareness	Does the initiative (i.e. the team) have appropriate mechanisms in place to facilitate sharing of information within the initiative (i.e. within the team) and are these used? (E.g. newsletters, workshops, reports available to all, results getting published, online information sharing platform……)<br><br>Scoring<br>Score between 0.0 (no sharing) and 1.0 (complete sharing	No mechanisms are in place among the sub-groups. Usual IT devices (chat, clouds, etc.) allows for data and info sharing	0.2
2. External mechanisms for sharing general information/awareness	Does the initiative have appropriate mechanisms in place to facilitate sharing of information outside the initiative and are these used? (E.g. newsletters, workshops, seminars/conferences, reports available to all, results getting published, online information sharing platform……)<br><br>Scoring<br>As above	Some groups of the team have in place initiatives to share data and information publicly ("Parliamone ora", UNAEUROPA)	0.5
3. Resources for sharing	Have resources been allocated to facilitate and ensure necessary data and information sharing?<br><br>Scoring<br>As above	The team as such has no specific fund for this task but some groups have specific resources, e.g. in EU project data sharing devices are almost compulsory	0.2
Data and information sharing			
4. Data/information quality	Are mechanisms/procedures in place to ensure data quality to allow sharing, e.g. data completeness, error-checking and correction of errors, clear and accurate descriptions of variables and of aggregations/calculations, documentation available.<br><br>Scoring<br>As above	Mechanisms to assure data quality within the team are usually in place in any research activity. Given the team structure, this does not concern the team as a whole but some groups which are used to work together, or individual groups . In EU project such mechanisms are part of the protocols (ROADMAP, …)	0.5
5. Data/information storage and accessibility	Are mechanisms/procedures in place to ensure safe and appropriate data storage (e.g. type of software, server, backup) with safe accessibility to facilitate sharing? (e.g. is extraction of data feasible without access without data managers, or are expert managers readily available for	The same situation as above. Data are shared within the team or within some groups (due to the team structure),	0.2

	extraction of data, is the process of data extraction bureaucratic/ cumbersome/overly time-consuming?)<br>Scoring<br>As above	especially in relation to specific projects. Usual IT devices (chat, clouds, etc.) or dedicated platform (in specific projects) allow for data storage	
6. Data/information sharing	Have appropriate (e.g. formal/written/signed) agreements been made concerning data sharing in the initiative? How well/how much are data being shared between people within the initiative? (e.g. compartmentalized<br>Scoring:<br>score 0-33), shared between few groups (34-66), fully shared between all actors in the initiative (score 67-100).	In general, there are no formal devices to share data within the team (due to the team structure), except in specific projects (see above).	0.2
Methods and results sharing			
7. Methods sharing	How well are methods shared between people within the initiative?<br>Scoring<br>compartmentalised (score 0-33), shared between few groups (34-66), fully shared between all in the initiative (score 67-100).	See answers to questions 4 and 5 above	0.5
8. Results sharing	How are results shared between people within the initiative?<br>Scoring:<br>compartmentalised (score 0-33), shared between few groups (34-66), fully shared between all in the initiative (score 67-100).	See answers to questions 4 and 5 above	0.5
Institutional memory/resilience			
9. Institutional memory	How well does the initiative include the creation or use of potential institutional knowledge reservoirs for data, methods and/or results over time? For example publications, detailed reports/manuals, database descriptions, standard operating procedures, introductions to inform new staff on essential procedures etc.<br>Scoring<br>0 = no institutional mechanism in place at all<br>1 = structured, fully accessible institutional mechanisms in place	Almost no mechanisms in place for this task. Usual mechanisms available at UNIBO are in place (institutional clouds, publication repository)	0.2
10. Resilience to change	Are mechanisms/procedures in place to safe-guard access to data, information and results in case of system change, e.g. change of IT-system, data ownership, institutional organizations.<br>Scoring<br>0 = no institutional mechanism in place at all	Usual mechanisms are in place at the institutional facilities (e.g. CESIA)	0.2

	1= specific, structured and codified mechanism are in place at the team level		

c. **Scoring and comments**

General information/awareness sharing	0.3
Data and information sharing	0.3
Methods and results sharing	0.5
Institutional memory/resilience	0.3
**Overall Sharing scoring**	**0.25**

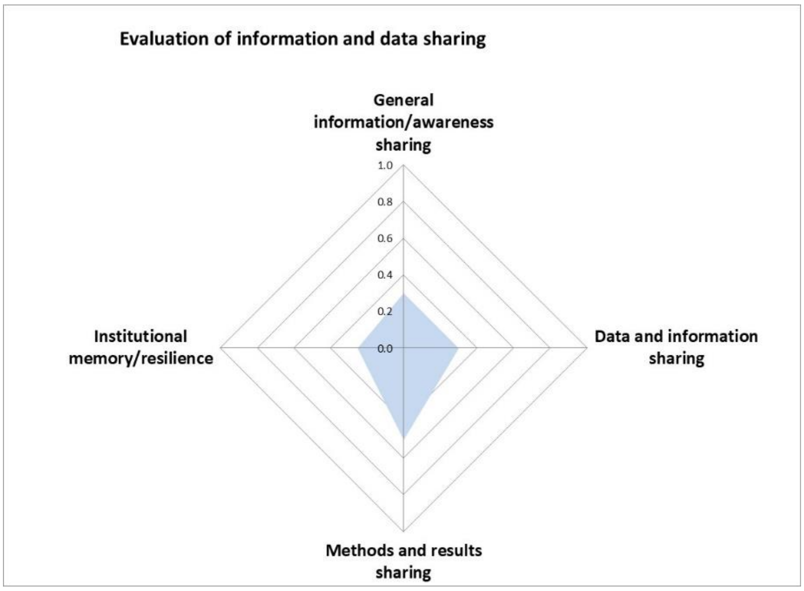

5.5. Learning

Learning is a change in cognition, potential or actual behaviour through better knowledge and understanding (Ruëgg, Häsler and Zinsstag, 2018). An institution or a team confronted with complex problems can evolve and gain effectiveness if it incorporates new knowledge and translate it into behaviour and operation in all its parts or members. It is thus desirable the that a process of mutual learning is widespread among team members and between the team, stakeholders and the external environment. On the other side, a mutual learning process should be the outcome of inter-/trans-disciplinary approach leading to a better understanding of complexity based on system thinking. In practice, learning may occur at individual level, as the result of an occasional, face-to -face process, with scarce impact on behaviours and team practices; or it may involve the team as a whole on the basis of a systematic protocols, encoded in institutional practices with strong feedback on team behaviour. In this section we try to evaluate what is the level of learning occurring within the team, what kind of organization, protocols or facilities are in place to serve this aim, to what aims it is directed (e.g. modifying individual behaviours, changing team routines, or affirm values and paradigms). To do this we distinguish **3 levels of learning**, respectively: - individual level, - team level and - organization level; and **3 types of learning**, respectively:

- Basic learning: information is circulated among the team but does not end into practice
- Adaptive learning: information is circulated and applied in order to improve procedures
- Generative learning: information is circulated and applied to modify beliefs and norms of individuals

In order to evaluate the degree of learning occurring in the environment where the initiative is implemented, we further distinguish **2 more learning levels**, respectively:

- the direct environment learning: this level focuses on the learning occurring between the team and the actors and stakeholders of the initiative
- the general environment learning: this level focuses on the learning occurring between the team and the cultural and socio-economic context of the initiative.

These two levels of learning are assumed to influence only adaptive and generative learning. Table 5.13 below resumes learning levels and types

Table 5.13 – Learning levels and types

Learning levels	Learning types		
	**Basic learning**	**Adaptive learning**	**Generative learning**
Individual	Information is circulated but does not end into practice	Information is applied to improve procedures	Information modifies beliefs and norms of individuals
Team	Teams meet to exchange information	Teams meet and discuss to find the best view to support decision making	Teams face and analyse complex issues to produce new ideas, views or approaches.
Organization	Information and knowledge are collected and stored	Collected information is discussed and acted upon at various levels within the organisation	Information leads to change in fundamentals and objectives of the organisation
Direct environment	*(Actors, stakeholders of the OH initiative)*	Actors and stakeholders support adaptive learning to improve existing procedures, processes, competences and technologies	Actors and stakeholders support generative learning, i.e. questioning the existing norms and encouraging new vision
General environment	*(Culture, economics, political situation surrounding the OH initiative)*	The general environment supports adaptive learning, to improve existing procedures, processes, competences and technologies	The general environment supports generative learning i.e questioning the existing norms and encouraging vision

a. Scoring questions

The questionnaire is based on 13 questions grouped in 5 levels which reflect the learning levels mentioned above (individual, team, organizational, direct and general environment) (Table 5.14 below). Each level is articulated according the types of learning (basic, adaptive and generative).

Table 5.14 – Attributes of Learning

Individual learning	1-3
Team learning	4-6
Organisational learning	7-9
Direct learning	10-11
General environment learning	12-13

Scoring is based on the assumption that, for each learning level, generative learning is assigned the greater importance (higher score) while basic learning is assigned the lower importance.[4]

[4] This translates in a different weight assigned to basic learning (0.10), adaptive learning (0.30) and generative learning (0.6).

The evaluation requires that the statements of the second column of Table 5.15 are judged according to a five grades Likert scale. Grades are: Always - Very often – Sometimes – Rarely – Never.

Table 5.15 – Scoring questions: Learning

Questions	Statement	Supporting reasoning	Score
Individual learning			
1. Basic learning	Individuals receive information which may be understood and may potentially lead to learning, but it is not put into practice in or outside the initiative by the individuals		Sometimes
2. Adaptive Learning	Information is understood, learnt and applied to improve procedures, competencies, technologies and paradigms without challenging the individuals' underlying beliefs and assumptions		Sometimes
3. Generative Learning	Information is understood and learnt by individuals and applied to improve procedures, competencies, technologies and paradigms as a result of modified underlying beliefs and norms of individuals		Sometimes
Team learning			
4. Basic learning	Teams meet to exchange information for reporting purposes		Sometimes
5. Adaptive Learning	When teams meet different views are presented, defended and discussed to find the best view to support decision making		Sometimes
6. Generative Learning	When teams meet complex issues are explored through dissection of views and assumptions of team members resulting in a move towards building new ideas, view or approach.		Sometimes
Organizational learning			
7. Basic Learning	Existing/circulating information and knowledge is collected and stored		Rarely
8. Adaptive Learning	Collected information is shared and discussed and acted upon at various levels within the organization(s)		Rarely
9. Generative Learning	Collected information is shared and discussed and leads to change in fundamentals and objectives across all levels within the organization(s)		Rarely
Direct environment			
10. For Adaptive learning	The direct environment of the OH initiative (stakeholders involved) is/was supportive for adaptive learning, i.e. learning that focuses on correcting or improving existing procedures, processes, competences and technologies		Sometimes
11. For Generative learning	The direct environment of the OH initiative (stakeholders involved) is/was supportive for generative learning, i.e. learning that focuses on questioning the existing norms and that encourages to see beyond the existing situation		Rarely

General environment			
**12.** For adaptive learning	The general environment (e.g. culture, economics, political situation) surrounding the OH initiative is/was supportive for adaptive learning, i.e. learning that focuses on correcting or improving existing procedures, processes, competences and technologies		Sometimes
**13.** For Generative learning	The general environment (e.g. culture, economics, political situation) surrounding the OH initiative is/was supportive for generative learning, i.e. learning that focuses on questioning the existing norms and that encourages to see beyond the existing situation		Sometimes

b. Scoring and comments

Focus on adaptive and generative individual learning	0.5
Focus on adaptive and generative team learning	0.5
Focus on adaptive and generative organisational learning	0.3
Direct learning environment supportive of adaptive and generative learning	0.3
General learning environment supportive of adaptive and generative learning	0.5
**Overall Learning score**	**0.42**

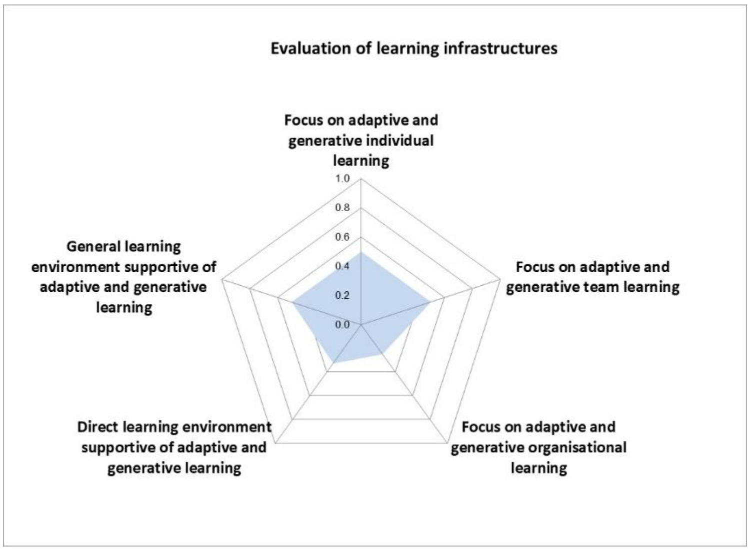

5.6. Systemic organization

Systemic organization dimension tries to assess the type of management and leadership of the team. It is assumed that a OH oriented management should be able to involve competent members in relation to problems faced by the team and to specific aims, support team working, solve potential conflicts or critical situations, be flexible, open to criticisms and innovation, adaptive to changes determined by the internal and external environment

a. Scoring questions

Scoring questions are structured as shown in Table 5.16 below

Table 5.16 - Attributes of Systemic organization

Team structures	1-7
Leadership style	8-12
Competence	13-14
Innovation and OH orientation	15-17

Table 5.17 – Scoring questions: Systemic organization

Questions	Focus of the question	Supporting reasoning	Score
Team structures			
1. How is teamwork implemented in this initiative?	The question focuses on whether the initiative is based on teamwork. The question assumes that if the initiative shows teamwork across disciplines, this is a sign of high inter- and trans-disciplinary quality. Describe any measures taken to encourage teamwork.<br>Scoring<br>Score the teamwork across disciplines on a scale from 0 to 1, with 0 no teamwork, 1 well-functioning teamwork.	Due to team structure, teamwork is regularly applied in each sub-group, and among some sub-groups. As sub-groups have disciplinary features, the above-mentioned situation also influences teamwork in the team as a whole	0.5
2. If more teams than one are mentioned, how good are the inter-team relations?	The question probes for how well different teams work together for the overall aim of the solving the problem. The question assumes that if the initiative shows good inter-team relations, this is a sign of high inter- and transdisciplinary quality.<br>Scoring<br>Please score the inter-team relations on a scale from 0 to 1 with 0 competition, 1 cooperation. If only one team in the initiative keep cell empty.	See above. Due to team structure, relationships among groups are regularly applied in some sub-groups but it's not a general feature of the team.	0.5
3. Do all teams have clear objectives?	The question determines whether the team/s are well structured or not?<br>Scoring<br>Score on a scale from 0 to 1, with 0 no clear objectives, 1 clear objective. For several teams use the average	In general, the team has clear enough objectives, though sometimes this happens on an individual (say sub-groups) basis (i.e. inside a group)	0.6
4. How closely do team members work together to achieve the teams' objectives?	The question determines whether the team/s are well structured or not?<br>Scoring<br>Score on a scale from 0 to 1, with 0 no teamwork, 1=close collaboration. For several teams use the average.	In general, team members work very closely, though sometimes this happens on an individual basis (i.e. inside a group)	0.6

5. How clearly are the roles differentiated for team members within the team?	The question determines whether the team/s are well structured or not?<br>Scoring<br>Score on a scale from 0 to 1, with 0 no clarity about roles, confusion; 1 clear roles. For several teams use the average	Due to the structure of the team, roles are in general well structured within the sub-groups and among the sub-groups in some cases but not in the team as a whole	0.4
6. Are the teams recognized by the community/department/s/official organizations as clearly defined team(s)?	The question determines whether the team/s are well structured or not?<br>Scoring<br>Score on a scale from 0 to 1, with 0 no recognition, 1 recognition, appreciation and respect. For several teams use the average	Due to the structure of the team, the team as such has no recognition. Some groups are well recognized in the environment	0.4
7. How frequently did the teams meet to discuss their effectiveness and how it could be improved?	The question determines whether the team/s are well structured or not?<br>Scoring<br>Score the frequency on a scale from 0 to 1, with 0 never, 1 frequently	Due to the structure of the team, meeting occur frequently among some groups but not at team level	0.6
Management			
8. How well do the management structures match and support the initiative's goal and combination of disciplines and fields of expertise?	Describe and evaluate the management structures involved in this initiative. It is assumed that an elaborated management structure is a sign of high inter- and transdisciplinary quality.<br>Scoring<br>Score the aptitude of the management structure to support the goals and trans-disciplinarity on a scale of 0 to 1, with 0 inappropriate structure, 1 well fit structure	Due to the structure of the team management structures are not defined for the team as a whole. For more integrated groups management recognition is almost informal, though effective in supporting initiative's goals	0.6
9. How would you characterize the leadership in the initiative in regard to task-orientation, relationship-orientation and change-orientation?	What is the type of leadership demonstrated in the initiative? E.g.: Small and collated (Single leader, central leader, informal connections, face-to-face processes, teambuilding, leader needs process skill); Large and dispersed (Multiple leaders/champions, leaders in brokerage positions, coordination needed among leaders, leaders as translators and conflict handlers).<br>Scoring<br>Score the leadership on a scale from 0 to 1 with 0 for no leadership, 0.1 a focus on task-orientation, 0.3 a focus on relationship-orientation, 0.5 a focus on change-orientation, 0.7 some of all, but unbalanced, 1 a well-balanced combination	Leadership supports in complying with tasks, relationship and change. Due to the structure of the team, there is a mix of central leadership (a leader inspiring and promoting several activities) and decentralized leadership at the level of the individual groups. Mixing the above-mentioned aspects (effective leadership in different directions but with almost informal means, dispersed among groups) we consider a moderate positive score	0.6

10. Does the initiative demonstrate open mindedness?	Score the open-mindedness on a scale from 0 to 1, with 0 self-centered atmosphere, 1 strong extroversion.	Yes, all the team members and group leaders show open mindedness	0.8
11. Does the initiative demonstrate changing hierarchies?	Hierarchical structures might prevail on needed changes to provide solutions to OH-challenges. Does the initiative allow for changing such hierarchies?<br>Scoring<br>Score the change of hierarchies on a scale from 0 to 1, with 0 static hierarchy, 1 hierarchy adapts to situation at hand.	No hierarchical structures prevail on changes	0.8
12. Does the initiative demonstrate ability to bear and manage tensions?	The question assumes that if the initiative shows core values of inter- and transdisciplinary ethics, this is a sign of high inter- and transdisciplinary quality.<br>Scoring<br>Score the ability on a scale from 0 to 1, with 0 no ability, 1 high ability	Yes, in general in all groups	0.8
Actors and Competences			
13. How well do/did the disciplinary composition and the competence in the team(s) permit the working towards the essential aspects of their objectives?	The question focuses on identification of inter-/trans-disciplinarity in the team/s.<br>Scoring<br>Score on a scale from 0 to 1, with 0 inappropriate team composition in terms of competence, 1 perfect match of competences and team roles. For several teams use the average	Competences are adequate to work toward objectives, given the team structure	0.7
14. Are the competences displayed by the various disciplines appropriate to the problem and its solution (relevant knowledge applied, roles in the initiative, possibilities for implementing results)?	The question checks for competences of the different disciplines and whether these competences are relevant to the problem<br>Scoring<br>Score on a scale from 0 to 1, with 0 inappropriate team composition for the problem, 1 perfect match of team roles to solve problem efficiently and effectively. For several teams use the average	As above, given the team structure	0.7
OH focus and innovation			
15. Is the One Health challenge adequately translated into scientific or developmental questions?	The question checks whether the scientific questions raised, as well as methodologies probes for contribution to new knowledge<br>Scoring<br>Score on a scale from 0 to 1, with 0 not well translated, 1 good translation	Yes, they do	0.8

16. Is the current state of knowledge taken into consideration (including information about relevant societal issues and structures)?	Scoring<br>Score on a scale from 0 to 1, with 0 not considered, 1 completely integrated	Yes it is	0.8
17. Did the initiative provide relevant innovation in relation to the state of knowledge and the OH challenge?	Evaluate how the initiative tries/tried out or provided new solutions/approaches to a challenge that could not be solved using previously tried / conventional methods<br><br>Scoring<br>Score the relevance on a scale from 0 to 1 with 0 not relevant, 1 highly relevant	Yes	0.8

b. Scoring and comments

Team structures	0.5
Management & leadership	0.72
Competence	0.8
Focus and innovation	0.7
**Overall Systemic org. Score**	**0.6**

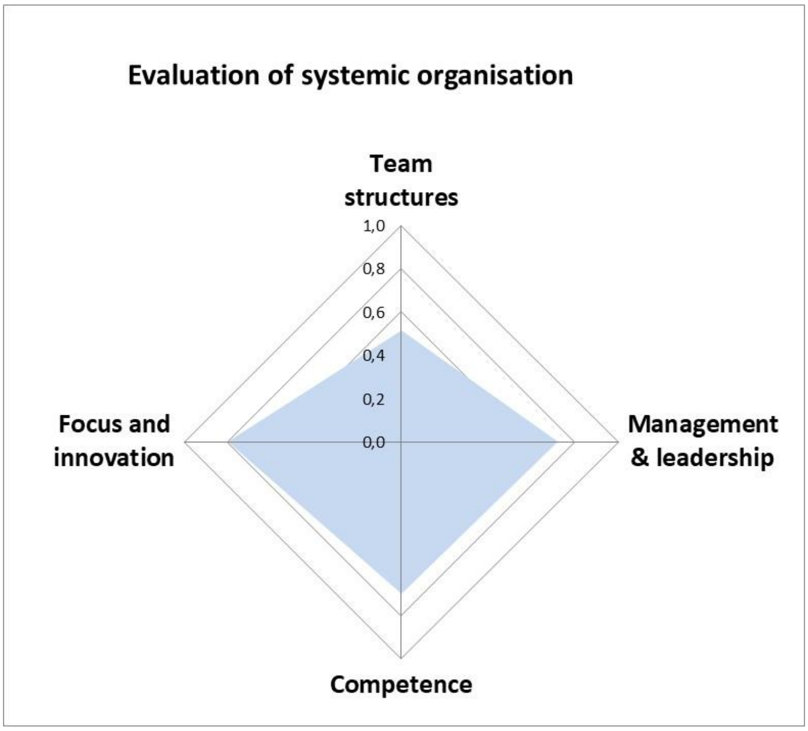

5.7. OH evaluation

OH operation	Thinking (ScT)	0.60

	Planning (ScP)	0.30
	Working (ScW)	0.70
Supporting infrastructure	Sharing (ScS)	0.25
	Learning (ScL)	0.42
	Systemic organisation (ScO)	0.60

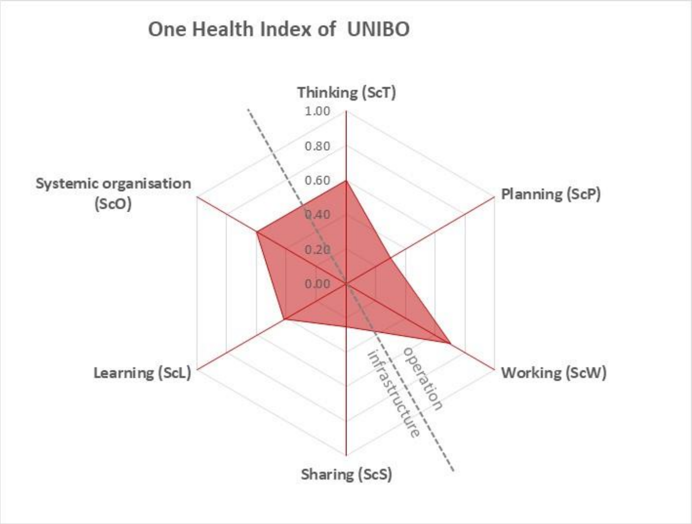

Supplement: Supplementary file 2 [file Data_Sheet_1.pdf]
